# High HIV incidence among young women in South Africa: Data from a large prospective study

**DOI:** 10.1371/journal.pone.0269317

**Published:** 2022-06-03

**Authors:** Thesla Palanee-Phillips, Helen V. Rees, Kate B. Heller, Khatija Ahmed, Joanne Batting, Ivana Beesham, Renee Heffron, Jessica Justman, Heeran Makkan, Timothy D. Mastro, Susan A. Morrison, Nelly Mugo, Gonasagrie Nair, James Kiarie, Neena M. Philip, Melanie Pleaner, Krishnaveni Reddy, Pearl Selepe, Petrus S. Steyn, Caitlin W. Scoville, Jenni Smit, Katherine K. Thomas, Deborah Donnell, Jared M. Baeten, for the ECHO Trial Consortium

**Affiliations:** 1 Wits Reproductive Health and HIV Institute (Wits RHI), University of the Witwatersrand, Faculty of Health Sciences, Johannesburg, South Africa; 2 Departments of Global Health, Medicine and Epidemiology, University of Washington, Seattle, Washington, United States of America; 3 Setshaba Research Centre, Soshanguve, South Africa; 4 Effective Care Research Unit (ECRU), University of the Witwatersrand/Fort Hare, East London, South Africa; 5 Department of Obstetrics and Gynaecology, Faculty of Health Sciences, University of the Witwatersrand, Durban, South Africa; 6 Mailman School of Public Health, ICAP at Columbia University, New York, New York, United States of America; 7 Klerksdorp Clinical Research Centre, The Aurum Institute, Johannesburg, South Africa; 8 FHI 360, Durham, North Carolina, United States of America; 9 Kenya Medical Research Institute (KEMRI), Nairobi, Kenya; 10 Emavundleni Research Centre, University of Cape Town, Cape Town, South Africa; 11 Department of Medicine, Faculty of Medicine and Health Sciences, Centre for Medical Ethics and Law, Stellenbosch University, Stellenbosch, South Africa; 12 UNDP-UNFPA-UNICEF-WHO-World Bank Special Programme of Research, Development and Research Training in Human Reproduction (HRP), World Health Organization, Geneva, Switzerland; 13 Gilead Sciences, Foster City, California, United States of America; University of KwaZulu-Natal, SOUTH AFRICA

## Abstract

**Introduction:**

South Africa has the highest national burden of HIV globally. Understanding drivers of HIV acquisition in recently completed, prospective studies in which HIV was an endpoint may help inform the strategy and investments in national HIV prevention efforts and guide the design of future HIV prevention trials. We assessed HIV incidence and correlates of incidence among women enrolled in ECHO (Evidence for Contraceptive Options and HIV Outcomes), a large, open-label randomized clinical trial that compared three highly effective. reversible methods of contraception and rates of HIV acquisition.

**Methods:**

During December 2015 to October 2018, ECHO followed sexually active, HIV-seronegative women, aged 16–35 years, seeking contraceptive services and willing to be randomized to one of three contraceptive methods (intramuscular depot medroxyprogesterone acetate, copper intrauterine device, or levonorgestrel implant) for 12–18 months at nine sites in South Africa. HIV incidence based on prospectively observed HIV seroconversion events. Cox proportional hazards regression models were used to define baseline cofactors related to incident HIV infection.

**Results:**

5768 women were enrolled and contributed 7647 woman-years of follow-up. The median age was 23 years and 62.5% were ≤24 years. A total of 345 incident HIV infections occurred, an incidence of 4.51 per 100 woman-years (95%CI 4.05–5.01). Incidence was >3 per 100 woman-years at all sites. Age ≤24 years, baseline infection with sexually transmitted infections, BMI≤30, and having new or multiple partners in the three months prior to enrollment were associated with incident HIV.

**Conclusions:**

HIV incidence was high among South African women seeking contraceptive services. Integration of diagnostic management of sexually transmitted infections alongside delivery of HIV prevention options in health facilities providing contraception services are needed to mitigate ongoing risks of HIV acquisition for this vulnerable population.

**Clinical trial registration:**

ClinicalTrials.gov, number NCT02550067 was the main Clinical Trial from which this secondary, non-randomized / observational analysis was derived with data limited to just South African sites.

## Introduction

South Africa has the largest national HIV epidemic globally, with an estimated 7.9 million persons living with HIV [1]. In this endemic setting, the dominant mode of transmission is through heterosexual sex, and women account for over 60% of new infections [2]. Over the past decade, substantial investment has been made in South Africa to scale-up education on and access to HIV testing, antiretroviral therapy and oral pre-exposure prophylaxis (PrEP), including the implementation of universal test and treat (UTT). The 2017 South African National household-based HIV Prevalence, Incidence, Behaviour and Communication Survey estimated HIV prevalence among adults aged 15 to 49 years to be 26.3% among females and 14.8% among males. HIV incidence was higher in the younger age groups (1.51% per year among those 15–24 years). Notably, that survey also estimated that national annualized HIV incidence among adults, measured with a cross-sectional algorithm including LAg-Avidity assay, was lower than in the previous national survey done in 2012 by the same group: 0.93% among females and 0.69% among males [1].

Based on their historically high rates of HIV, much of the focus of HIV prevention and care efforts in South Africa has been concentrated on women. It has been anticipated that scale-up of HIV testing, extensive access to antiretroviral therapy, and primary prevention services, including expanded PrEP access to populations including adolescent girls and young women (AGYW), would lead to a national decrease in HIV incidence. South Africa has made significant progress towards the UNAIDS 90–90–90 targets [3]. Improvements in HIV testing, increasing awareness of HIV status, and improved treatment of people living with HIV are encouraging. Medical male circumcision has increased significantly since 2012. Nevertheless, more work is needed amongst young women who remain among the most affected, particularly as strategies to reduce infectiousness such as HIV treatment and condom use require action by male partners. Further needed are efforts to address the impact of associated social factors, such as age-disparate relationships, inconsistent condom use and early sexual debut in increased risk [3].

Notably, although the epidemic is generalized in South Africa, it varies significantly across and within different geographies. Research has shown that people living in informal areas of the country continue to be most-at-risk for HIV, with a higher HIV incidence than people in other areas. This is likely linked to limited access to effective HIV prevention and treatment strategies over time in informal areas as well as the direct impact on HIV incidence trajectories in these settings. These findings suggests that a strong multi-sectoral approach is necessary to address socioeconomic challenges that continue to fuel the epidemic [3]. Contextual understanding of the HIV epidemic is critical to develop and implement suitable, effective HIV interventions in these settings.

Prospective, cohort-based surveys offer important, directly-measured data on HIV incidence and risk factors for incident HIV [4]. They have been used since the beginning of the epidemic to understand HIV risk in specific populations, inform national-level programme planning and design of future HIV prevention clinical trials. We conducted the Evidence for Contraceptive Options and HIV Outcomes (ECHO) Trial, a prospective open- label clinical trial among women in four southern and east African countries during December 2015 to October 2018. We present data here on HIV incidence from the participating sites in South Africa, offering an opportunity to better understand near-contemporary HIV incidence and factors associated with acquisition for women living across a range of geographies in South Africa.

## Methods

The ECHO Trial was a multicentre, open-label, randomized trial of 7,829 HIV-seronegative women seeking effective contraception in Eswatini, Kenya, South Africa, and Zambia conducted from 2015–2018 (Clinicaltrials.gov NCT02550067); detailed trial methods and results have been published previously [5, 6]. Among the twelve ECHO sites, data from the nine South African sites were included in the present analysis.

### Study design, participants and ethics

Briefly, women were invited to enrol in the ECHO Trial from December 2015 through September 2017 [6]. Women who were HIV-seronegative, aged 16–35 years, seeking effective contraception, without medical contraindications to the trial contraceptive methods, willing to use the assigned method for 18 months, reported not using injectable, intrauterine, or implantable contraception for the previous six months, reporting being sexually active and not pregnant, were enrolled. Where the potential to enrol minors <18 years was an option, parents or legal guardians provided written informed consent and the minors provided written informed assent; South African IRBs did not permit waiver of consent.

At enrolment, women were randomly assigned (1:1:1) to intramuscular depot medroxyprogesterone acetate (DMPA-IM), copper intrauterine device (IUD), or levonorgestrel (LNG) implant. Women returned for scheduled follow-up visits every three months for up to eighteen months, and visits included HIV serological testing, contraceptive counselling, clinical safety monitoring, and syndromic STI management. In addition, laboratory testing for *Neisseria gonorrhoeae* and *Chlamydia trachomatis* using the Cepheid GeneXpert and Abbott Realtime platforms, and herpes simplex virus type 2 (HSV-2) using the FOCUS HerpeSelect 2 ELISA IgG testing platform were conducted at baseline and repeated at possible seroconversion and final study visit [6].

At every visit, participants received a comprehensive package of HIV prevention services including HIV risk reduction counselling, participant and partner HIV testing and syndromic STI management, ART referrals, and condoms; HIV pre-exposure prophylaxis (PrEP) was provided on-site late into the study, as it became a part of national standard of care [6–8]. Counselling messages related to HIV risk reduction, including PrEP and condom use, were designed and implemented consistently across the three randomized groups throughout the trial [6]. The trial was implemented in accordance with the Declaration of Helsinki and Good Clinical Practice standards.

Institutional review boards at each site approved the study protocol, in addition to an overall study IRB, and all women provided written informed consent. Specifically, overall review was provided by the FHI 360 Protection of Human Subjects Committee. For the South African sites, the University of Witwatersrand Human Research Ethics Committee, University of Cape Town Human Research Ethics Committee provided approval.

### HIV testing and outcomes

Incident HIV infection was identified using dual parallel rapid testing. Sites used two of the following assays: Alere ABON HIV 1/2/O Tri-Line, Advanced Quality ONE STEP Anti-HIV (1&2), BioTracer HIV-1/2, Determine HIV-1/2, First Response HIV 1–2, OraQuick Advance HIV-1/2, Uni-Gold Recombigen HIV-1/2. Positive or indeterminate rapid test results were confirmed by Enzyme linked Immunosorbent Assay (EIA) on the Abbott ARCHITECT platform and HIV RNA PCR on the Abbott Realtime platform, additional testing as needed according to a standard HIV testing algorithm, and confirmed by an endpoints committee. For women testing HIV seropositive, we assessed archived plasma samples from the enrolment visit using HIV RNA PCR and excluded those with detectable HIV RNA.

For those completing follow-up as HIV seronegative, follow-up time was defined as time accrued in days from study enrolment to the last HIV test result; for those acquiring HIV, time was accrued in days from study enrolment to the first HIV test indicating possible HIV infection confirmed by the study HIV testing algorithm.

#### Statistical analysis

HIV sero-incidence rates based on prospectively observed HIV seroconversions and exact 95% CIs based on a Poisson distributions assumption were assessed, reported overall and for each site.

Cox proportional hazards regression models were used to define cofactors related to incident HIV, adjusted for randomized group and stratified by site. Analyses were limited to baseline variables, with the goal of understanding factors that might predict HIV acquisition over the near 12–18 months when women were seen in a one-time visit. Baseline factors included body mass index (BMI) (≤30 versus >30 kg/m^2^), age (≤ 24 years versus > 24 years), coital frequency in previous three months (≤median versus > median), having living children (0 versus 1 or more), living with husband or primary partner (no versus yes), vaginal sex without a condom in the previous three months (ever versus never), more than one sex partner or a new sex partner in the previous three months (yes versus no), HSV-2 status (positive versus negative), *N*. *gonorrhoeae* and/or *C*. *trachomatis* infection (any positive versus both negative). Separate Cox proportional hazards regression models were conducted for each baseline cofactor and included a three-way variable for randomized treatment arm, stratified by site. Parameter estimates from the Cox models were used to calculate Z-scores against the null hypothesis of HR = 1.0 and corresponding two-sided p-values. The full multivariable model was stratified by site and includes randomized treatment arm along with the baseline cofactors. Analyses were completed using SAS, version 9.4.

## Result

### Analytical sample

Of 7829 participants in ECHO, 5768 were enrolled at the nine sites located in South Africa. Eleven women determined to be infected at enrolment were excluded from analyses, as were 87 women who never contributed a follow-up HIV test. The remaining 5670 women contributed 7647 woman-years of follow-up (Table 1).

**Table 1:** Baseline socio-demographic, behavioural characteristics and HIV seroincidence—Individual and multivariable model results.

Characteristic	Category	Enrolled Sample size	Contributed follow-up ^β^ (n = 5670)	# HIV seroconversions / # Woman-Years of Follow-up (incidence per 100 woman-years)	Univariable models ^#^				Multivariable model ^#^			
					Hazard Ratio	95% CI Lower bound	95% CI Upper bound	p-value	Hazard ratio	95% CI Lower bound	95% CI Upper bound	p-value
**Age (years)**	Median (IQR)	23 (20, 26)										
**Age (years)**	≤24	3603 (62.5%)	3535 (62.3%)	245 / 4749 (5.16)	1.37	1.08	1.73	0.010	1.33	1.01	1.77	0.046
	>24	2165 (37.5%)	2135 (37.6%)	100 / 2898 (3.45)	Ref							
**BMI (kg/m** ^**2**^ **)**	≤30	3938 (68.3%)	3869 (68.2%)	256/5203 (4.92)	1.34	1.05	1.71	0.018	1.33	1.01	1.74	0.041
	>30	1826 (31.7%)	1797 (31.7%)	89/2439 (3.65)	Ref							
**Gravidity**	Gravid	4398 (76.2%)	4335 (76.5%)	255/5851 (4.36)	Ref							
	Nulligravid	1370 (23.8%)	1335 (23.5%)	90/1796 (5.01)	1.11	0.87	1.41	0.415	1.30	0.69	2.46	0.414
**Living Children**	None	1605 (27.8%)	1565 (27.6%)	102/2110 (4.83)	1.03	0.81	1.31	0.790	0.72	0.39	1.34	0.302
	At least one	4163 (72.2%)	4105 (72.4%)	243/5537 (4.39)	Ref							
**Living with Partner**	No	4856 (84.2%)	4769 (84.1%)	315/6415 (4.91)	1.74	1.19	2.57	0.005	1.41	0.93	2.13	0.105
	Yes	912 (15.8%)	901 (15.9%)	30/1232 (2.44)	Ref							
**Unprotected Sex** ^!^	Never	1556 (27.0%)	1528 (26.9%)	91/2059 (4.42)	Ref							
	Sometimes-always	4211 (73.0%)	4141 (73.0%)	254/5587 (4.55)	1.04	0.81	1.32	0.770	1.03	0.79	1.34	0.832
**New or Multiple Partners** ^$^	No	5209 (90.3%)	5125 (90.4%)	294/6930 (4.24)	Ref							
	Yes	553 (9.6%)	539 (9.5%)	51/711 (7.18)	1.68	1.24	2.27	< .001	1.62	1.18	2.24	0.003
**Coital Acts** ^$^	≤median (9)	2990 (51.8%)	2940 (51.9%)	206/3941 (5.23)	1.25	1.00	1.57	0.048	1.17	0.92	1.50	0.206
	>median (9)	2777 (48.1%)	2729 (48.1%)	139/3704 (3.75)	Ref							
***N*. *gonorrhoeae* or *C*. *trachomatis***	Both negative	4367 (75.7%)	4294 (75.7%)	235/5815 (4.04)	Ref							
	Either/both positive	1389 (24.1%)	1364 (24.1%)	109/1815 (6.00)	1.49	1.18	1.87	< .001	1.42	1.11	1.81	0.006
**HSV-2 Serology**	Negative	2874 (49.8%)	2825 (49.8%)	149/3824 (3.90)	Ref							
	Positive	2226 (38.6%)	2188 (38.9%)	151/2941 (5.13)	1.35	1.07	1.70	0.010	1.48	1.16	1.89	0.001

^!^Never unprotected sex includes participants with no partner, no sex, or having sex but always using a condom in the previous three months. Any unprotected sex includes participants who had sex at least once and never, rarely, sometimes, or often used a condom in the previous three months.

^$^In 3 months prior to enrolment.

NOTE: HSV-2 EIA results are classified as follows: <0.90 = Negative, 0.90 to 3.50 = Indeterminate, >3.50 = Positive

^β^ Median age 23, IQR (20,26). Four participants are missing data on BMI (total 0.07%). One participant is missing data on unprotected sex and coital acts. Six participants (total 0.11%) are missing data on new sex partners in the previous 3 months. 12 participants are missing data on *N*. *gonorrhoeae* and *C*. *trachomatis* (total 0.2%). N = 657 unknown or indeterminate HSV-2 results excluded.

^#^ Separate Cox PH models for each cofactor adjusted for randomized arm and stratified by site. Multivariable model includes all baseline cofactors.

The median age was 23 (interquartile range 20, 26) years and 62.5% of participants were ≤24 years of age, 28.3% were between 25–30 years and 9.3% between 31–35 years. No minors were enrolled in South Africa. The majority of participants (68.3%) had a BMI ≤30 and 72.2% reported at least one living child. Only 15.8% of participants reported living with their partner and 9.6% reported having new or multiple partners in the three months prior to enrolment. Nearly three-quarters (73%) engaged in some condomless sex in the previous three months. Baseline STI infection with either *N*. *gonorrhoeae* or *C*. *trachomatis* was detected among 24.1% of participants and HSV-2 was detected among 38.6%.

The decision to use the categorizations for *N*. *gonorrhoeae* or *C*. *trachomatis* in Table 1 were for ease of interpretation alongside the primary manuscript [6] where these subgroups were also used. Baseline prevalence of *N*. *gonorrhoeae* was relatively low compared to *C*. *trachomatis* in the ECHO sites in South Africa (range 3.4–8.6% for *N*. *gonorrhoeae* and 18.4–28.2% *C*. *trachomatis*). In addition, in the overall ECHO study population, 4.6% reported a new partner in the 3 months prior to enrollment, and 6.8% reported more than 1 partner in the prior 3 months so the new and more than 1 partner categories were combined as well (Table 1).

**Fig 1:**
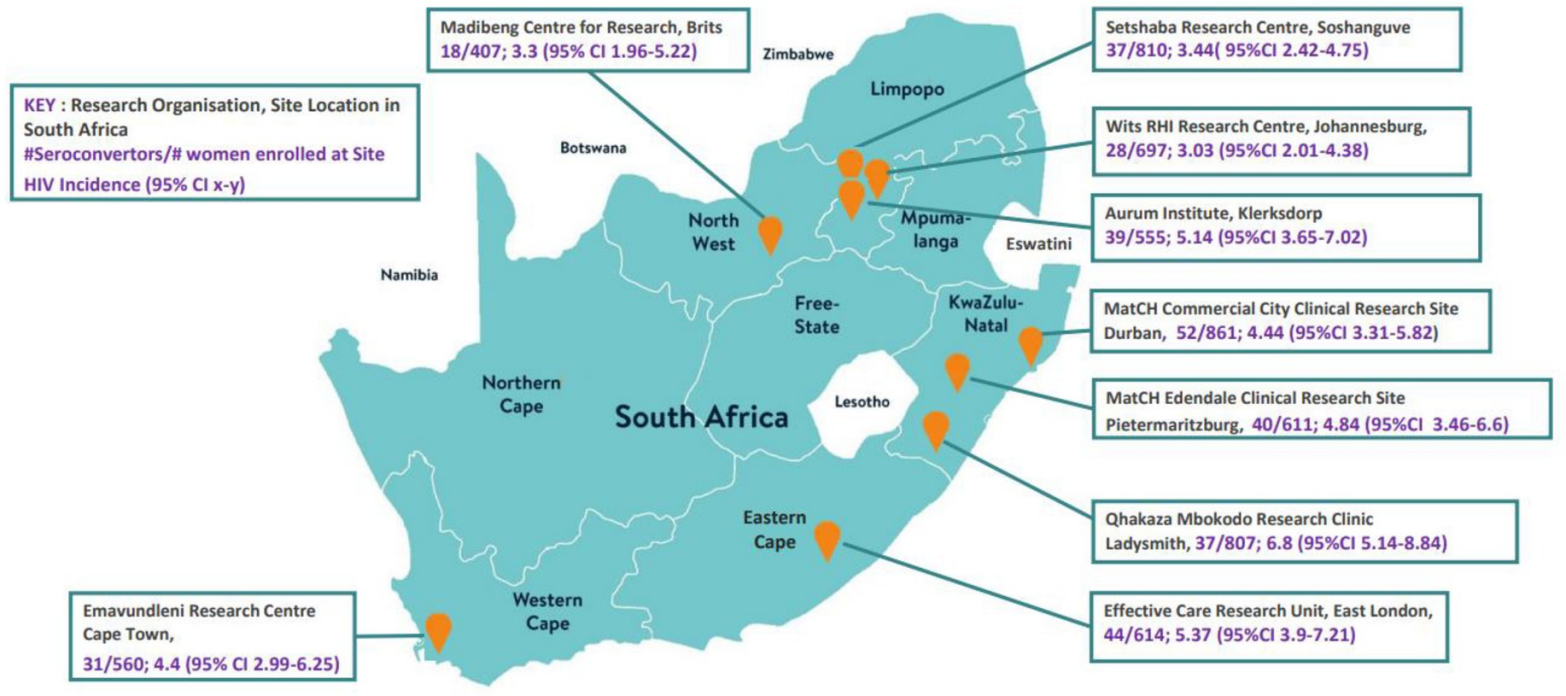
HIV seroincidence by South African ECHO Trial Site between December 2015 to October 2018.

A total of 345 incident HIV infections occurred, for an HIV incidence of 4.51 per 100 woman-years (95% CI 4.05–5.01). Incidence was >3 per 100 woman-years at all sites, the highest being 6.80 per 100 woman-years (95% CI 5.14–8.84) (Fig 1) at the Ladysmith site in KwaZulu-Natal followed by the East London site with incidence of 5.37 per 100 woman years (95% CI 3.9–7.21) and the Klerksdorp Site with incidence of 5.14 per 100 woman years (95%CI 3.65–7.02). These three sites were considered among the more informal area based sites. The remaining ECHO sites based in a range more peri-urban and urban locations in Johannesburg, Soshanguve, Brits, Durban and Cape Town all had incidence rates greater than 3 per 100 woman-years as shown in Fig 1.

Among South African women within the 18–20 year age range (n = 1493), HIV incidence per 100 woman years was 5.03 (95%CI 4.1–6.12) among those 21–30 years (n = 3470); and 4.72 (95% CI 4.13–5.36) and among those 31–35 years (n = 535). In the multivariable model, age ≤24 years, baseline infection with an STI (*N*. *gonorrhoeae* or *C*. *trachomatis* as well as HSV-2), BMI≤30, and having new or multiple partners were all strongly associated with HIV incidence (Table 1).

## Discussion

None of the three contraceptive methods that were evaluated in the ECHO trial were designed to be protective against HIV. Despite availability and provision of individualised HIV prevention care packages that included condoms, HIV counselling and testing as well as STI management throughout follow-up, compounded by years of substantial investment in HIV testing, treatment, and prevention generally in South Africa, HIV incidence was very high in this study, per WHO definitions of incidence among women at substantial risk of HIV infection [9]. These findings are important for ongoing programmatic efforts for curbing the HIV epidemic in women in South Africa and for the design of trials of new HIV prevention interventions.

Women were recruited for this trial on the basis of geography, being sexually active, and seeking pregnancy prevention, but not based on other drivers of HIV risk, such as transactional sex, history of STIs, or self-reported high-risk behaviours [6]. Thus, it is particularly concerning that HIV incidence was >4% per year overall and over 5% at three of these sites. Of note, recently-reported results from a completed HIV vaccine trial from South Africa reinforce our findings–in that study, HIV incidence among women was also >4% per year [10]. Moreover, our data support earlier research that has shown that people living in informal areas of the country continue to be at substantial risk for HIV [3]. Likely linked to limited access to HIV prevention and treatment strategies over time in informal areas as well as the direct impact on HIV incidence trajectories in these settings, more must be done to increase access to effective prevention and treatment services in these locations. Together, these data emphasize that women, particularly sexually active young women, remain highly vulnerable to HIV in South Africa. While the epidemic in South Africa may be generalised, it is not uniform, and our results are consistent with variation in the risk of HIV infection across the country as shown in the variable rates across the ECHO sites (Fig 1). Even within generalised epidemics, HIV is comprised of micro-epidemics in specific geographies and key populations, reflected in some variability in HIV incidence rates across sites in the ECHO trial. National HIV prevention efforts need frameworks and instruments to optimise the effect of available resources through strategic use of local epidemiological data.

Consistent condom use is one of the most effective prevention interventions for STIs, including HIV [11, 12]. Most participants (73%) reported engaging in some level of condomless sex. In South Africa, young women have been recognized as having particularly high HIV incidence [13, 14] and our data reinforce that younger women were at greater risk, likely reflecting both biological and economic vulnerability [13–15]. Having new or multiple partners was also strongly associated with HIV incidence, and concurrent partnerships have been increasingly prominent as known drivers of HIV transmission [16–21]. High baseline HSV-2, *N*. *gonorrhoeae* and *C*. *trachomatis* infection rates were seen and were strongly associated with increased risk of acquiring HIV. HIV and unmanaged STIs share a complex bidirectional relationship marked by increasing risk of HIV acquisition and transmission [22–26].

As new biomedical HIV prevention interventions are developed, tested, and prove efficacious, continuing to understand the drivers of high HIV incidence in populations will remain a priority. Some have proposed counterfactual or near-contemporaneous measures of HIV incidence as comparators, as use of placebo groups would violate ethical imperatives to provide access to the best available prevention methods, including PrEP [27–29]. Our results provide robust data for such comparisons.

## Conclusions

Young sexually active South African women of reproductive age continue to represent a priority population for HIV prevention interventions. Despite frequent counselling support paired with comprehensive HIV/STI prevention package, women remain at substantial risk with HIV incidence very high [9] among women enrolled in the ECHO Trial at all nine South African sites. Aggressive action directed towards provision of access to diverse, acceptable HIV prevention options combined with personalized risk reduction counselling are needed to mitigate the ongoing risk of HIV acquisition for this population. Additional results from the ECHO trial found that access to PrEP reduced HIV incidence substantially in this population [8] and efforts to expand PrEP services and PrEP options must be a priority. In addition, these findings underscore the critical importance of expanding HIV testing and rapid initiation of ART among HIV-infected men. Expansion of prevention and treatment options must include integration of HIV-specific biomedical, structural and behavioural interventions tailored to young women’s needs and social circumstances. Renewed efforts are required in communities and in public healthcare services to intensify the delivery of HIV interventions.

## Supporting information

S1 Protocol (PDF)
