# Supplementary material for: High HIV incidence among young women in South Africa: Data from a large prospective study
Source: PLoS One. 2022 Jun 3;17(6):e0269317. doi: 10.1371/journal.pone.0269317 (PMC9165791; doi:10.1371/journal.pone.0269317)
Supplement: S1 Protocol — (PDF) [file pone.0269317.s001.pdf]

**A Multi Center, Open-Label, Randomised Clinical Trial Comparing HIV Incidence and Contraceptive Benefits in Women using Depot Medroxyprogesterone Acetate (DMPA), Levonorgestrel (LNG) Implant, and Copper Intrauterine Devices (IUDs)**

**Short Title:** ***The Evidence for Contraceptive options and HIV Outcomes (ECHO) Trial***

**Version 5.0**

**Funding Agencies:** The Bill & Melinda Gates Foundation, the Swedish International Development Cooperation Agency (SIDA), and the Medical Research Council of South Africa. The U.S. Agency for International Development (USAID) and the South African government are donating contraceptives for the study.

**Clinical Trials.gov Identifier** NCT02550067

CONFIDENTIAL

## TABLE OF CONTENTS

ABBREVIATIONS AND ACRONYMS 1
CONSORTIUM GROUP TEAM MEMBERS* 3
INVESTIGATOR SIGNATURE FORM 5
PROTOCOL SUMMARY 6
1. KEY ROLES 8
1.1 ECHO Consortium 8
1.2 ECHO Structure 8
1.3 ECHO Organizational Roles 8

1.4 Protocol Identification 8
1.5 Sponsor Identification 9
1.6 Data Center 9
1.7 Study Operations Coordination 9
2. INTRODUCTION 9
2.1 Background and Rationale 9
2.2 Justification for a randomised clinical trial 10
2.3 Justification for three-arm design 11
2.4 Justification of choice of comparators 12
2.4.1 DMPA 12
2.4.2 Contraceptive implants 12
2.4.3 Copper IUD 13
2.5 Potential biological differences between different progestins 14
2.6 Contraceptive Continuation 14
3. OBJECTIVES AND ENDPOINTS 16
3.1 Primary Objective 16
3.2 Secondary Objectives 16
3.3 Tertiary Objectives 16
3.4 Primary Endpoint 16
3.5 Secondary Endpoints 16
3.6 Tertiary Endpoints 16
4. STUDY DESIGN 16
4.1 Trial Design 16
4.2 Study Setting 17
4.2.1 HIV prevalence and incidence in the proposed countries 17
4.3 Study Population 17
4.4 Eligibility Criteria 18
4.4.1 Inclusion Criteria 18
4.4.2 Exclusion Criteria 18
4.4.3 Inclusion of Adolescents 16-17 Years Old 19
4.5 Co-enrolment Criteria 19
4.6 Interventions 20

CONFIDENTIAL

4.6.1 Description/ Regimen of Study products 20
4.6.2 Administration/insertion of study products 20
4.6.3 Supply and accountability 21
4.6.4 Strategies for successful contraceptive initiation and assessing continuation 21
4.6.5 Contraception discontinuation 22
4.6.6 Concomitant Medications 22
5. STUDY PROCEDURES 22
5.1 Study Coordination 22
5.2 Recruitment/Pre-Screening 23
5.3 Study visit schedule 23
5.4 Clinical Evaluations, Procedures and Management 23
5.4.1 Medical, contraceptive, and reproductive history 23
5.4.2 Physical exam 24
5.4.3 Laboratory evaluations 24
5.4.4 Discontinuation of study product in response to observed adverse events 24
5.4.5 Sexually transmitted and reproductive tract infections and other clinical findings 25
5.4.6 Cervical Cancer Screening 25
5.4.7 Communicable Disease Reporting 25
5.4.8 HIV infection 25
5.4.9 Pregnancy 25
5.4.10 Criteria for early discontinuation of study participation 26
5.4.11 Management of discontinuation/changing of contraceptive method 26
5.5 Screening visit 26
5.6 Enrolment visit 27
5.7 Follow-up visits 28
5.7.1 One-month follow-up visit 28
5.7.2 Quarterly (3-monthly) follow-up visits 28
5.7.3 Final study visit 29
5.7.4 Possible HIV seroconversion visit 30
5.8 Unscheduled Visits 30
5.9 HIV Prevention Package 31
5.10 Primary HIV Endpoint Determination 31

CONFIDENTIAL

5.11 Specimen Collection and Processing 31
5.12 Specimen Handling and Biohazard Containment 31
5.13 Procedures for HIV Seroconverters and Ancillary Studies of Early HIV Disease 32
5.14 Procedures for ancillary studies of biologic correlates of HIV acquisition 32
5.15 Retention of study subjects 32
6. ASSIGNMENT OF INTERVENTIONS 32
6.1 Allocation 32
6.1.1 Sequence generation 33
6.1.2 Allocation concealment mechanism 33
6.1.3 Implementation 33
6.2 Blinding 33
7. DATA MANAGEMENT 33
7.1 Data collection methods 33
7.2 Data management responsibilities 33
8. STATISTICAL METHODS 34
8.1 Sample size 34
8.2 Statistical analyses 34
8.2.1 Primary analysis 35
8.2.2 Analysis of secondary endpoints 35
8.2.4 Methods for any additional analyses 36
8.2.4 Methods for handling missing data 36
8.2.5 Interim Analysis Plan Summary 37
9. MONITORING 37
9.1 Data monitoring: Data and Safety Monitoring Board 37
9.1.1 Monitoring Quality of Study Conduct Operational Characteristics and Implementation 37
9.1.2 Monitoring of Safety and HIV Acquisition Endpoints 38
9.2 Safety Monitoring by Protocol Team 38
9.2.1 Adverse Events (AEs) 38
9.2.2 Serious Adverse Events 39
9.2.3 Adverse Event Relationship to Study Product 39
9.2.4 Adverse Event Reporting Requirements 39
9.3 Social Harms Reporting 39

CONFIDENTIAL

9.4 Regulatory Requirements 40
9.4.1 Quality control and quality assurance 40
9.4.2. Source documents and access to source data/documents 40
9.5. Clinical Site monitoring 40
9.6 Auditing 41
10. GOOD PARTICIPATORY PRACTICE 41
11. ETHICS AND GENDER 42
11.1 Institutional Review Boards/ Ethics Committees 42
11.2 Protocol Registration 42
11.3 Risk Benefit Statement 42
11.3.1 Risks 42
11.3.2 Benefits 43
11.4 Informed Consent Process 44
11.4.1 Informed Consent for Minors 45
11.5 Participant Confidentiality 45
11.6 Compensation 45
11.7 Deception 45
11.8 Protocol Amendments 46
11.9 Declaration of interests 46
11.10 Ancillary and post-trial care 46
11.11 Gender Considerations 46
11.11.1 How the project responds to a public health need affecting men or women 46
11.11.2. How the research contributes to reducing inequities between women and men in health and health care 47
12. DISSEMINATION AND PUBLICATION POLICY 47
12.1 Applicability of Results 47
12.2 Communication of trial results 47
12.3 Access to data 48
12.4 Publication Policy 48
12.5 Authorship eligibility 48
13. MAIN PROBLEMS ANTICIPATED & PROPOSED SOLUTIONS 48
13.1 Main problems anticipated and proposed solutions 48
13.1.1 Recruitment 48

CONFIDENTIAL

13.1.2 Randomisation 49

13.1.3 Retention 49

14. REFERENCES 50

15. APPENDICES 53

Appendix 1: Membership and roles for the organizations comprising the ECHO Consortium 53

Appendix 2: ECHO Study Sites 54

Appendix 3: Three-arm study design: What will it answer? 55

Appendix 4: Advantages and Disadvantages of Specific Contraceptive Methods as a Study Arm 57

Appendix 5: Site Evaluation Metrics 59

Appendix 6: Schedule of study procedures 60

Appendix 7: HIV Testing Algorithm 62

Appendix 8: Additional sample collection for ancillary studies of biologic markers of early HIV disease 63

Appendix 9: Additional sample collection for ancillary studies of biologic correlates of HIV acquisition* 64

Appendix 10: Medications that may cause drug interactions with some study methods 66

Appendix 11: ECHO Governance 67

CONFIDENTIAL

**A Multi Center, Open-Label, Randomised Clinical Trial Comparing HIV Incidence and Contraceptive Benefits in Women using Depot Medroxyprogesterone Acetate (DMPA), Levonorgestrel (LNG) Implant, and Copper Intrauterine Devices (IUDs)**

## ABBREVIATIONS AND ACRONYMS

| | |
|---|---|
| AE | Adverse Event |
| ART | Antiretroviral Therapy |
| AVAC | AIDS Vaccine Advocacy Coalition |
| BMD | Bone Mineral Density |
| CAB | Community Advisory Board |
| COC | Combined Oral Contraceptive |
| CRF | Case Report Form |
| CT | *Chlamydia trachomatis* |
| DMPA | Depot medroxyprogesterone acetate |
| DSMB | Data and Safety Monitoring Board |
| EC | Ethics Committees |
| ECHO | Evidence for Contraceptive options and HIV Outcomes |
| ETG | Etonogestrel |
| FP | Family Planning |
| GC | *Neisseria gonorrhoeae* |
| GCP | Good Clinical Practices |
| GPP | Good Participatory Practice |
| GR | Glucocorticoid Receptor |
| HIV | Human Immunodeficiency Virus |
| IATA | International Air Transport Association |
| IC | Informed Consent |
| IM | Intramuscular |
| IRB | Institutional Review Board |
| IUD | Intrauterine Device |
| KEMRI | Kenya Medical Research Institute |
| LNG | Levonorgestrel |
| MEC | Medical Eligibility Criteria |
| MPA | Medroxyprogesterone acetate |
| NAAT | Nucleic Acid Amplification Test |
| NET-En | Norethisterone enanthate |

CONFIDENTIAL

| | |
|---|---|
| NIH | National Institutes of Health |
| NSAIDS | Nonsteroidal Anti-inflammatory Drugs |
| OHRP | Office for Human Research Protections |
| PH | Proportional Hazards |
| PHSC | Protection of Human Subjects Committee |
| PID | Pelvic Inflammatory Disease |
| POP | Progestin-only Pill |
| PR | Progesterone Receptor |
| QA/QC | Quality Assurance/Quality Control |
| RCT | Randomised Controlled Trial |
| RHR | Department of Reproductive Health Research |
| RP2 | Research Project Review Panel |
| SAE | Serious Adverse Event |
| SAP | Statistical Analysis Plan |
| SDMC | Statistical and Data Management Center |
| SOP | Standard Operating Procedure |
| SSP | Study Specific Procedure |
| STI | Sexually Transmitted Infection |
| UNAIDS | Joint United Nations Programme on HIV/AIDS |
| UW | University of Washington |
| VL | Viral Load |
| VTE | Venous Thromboembolism |
| WHO | World Health Organization |
| WRHI | Wits Reproductive Health & HIV Institute |
| w-y | woman years |

CONFIDENTIAL

**A Multi Center, Open-Label, Randomised Clinical Trial Comparing HIV Incidence and Contraceptive Benefits in Women using Depot Medroxyprogesterone Acetate (DMPA), Levonorgestrel (LNG) Implant, and Copper Intrauterine Devices (IUDs)**

## CONSORTIUM GROUP TEAM MEMBERS*

**FHI 360**
Timothy Mastro
Charles Morrison
Kavita Nanda
Douglas Taylor
Jennifer Deese
Irina Yacobson
Julia Welch
Florence Carayon-Lefebvre d'Hellencourt
Nikki Messenger
Nikki Walther
Maria Fawzy
Ginger Pittman
Pairin Seepolmuang
Tonya Colter
Colleen Macko

**Wits Reproductive Health & HIV Institute (**WRHI**)**
Helen Rees
Thesla Palanee
Deborah Baron
Clare Dott
Sinazo Pato
Nomthandazo Mbandazayo
Nkunda Vundamina
Melanie Pleaner
Reneilwe Boikanya
Daphne van der Wind

**University of Washington (UW) & Fred Hutchinson Cancer Research Center**
Jared Baeten
Connie Celum
Deborah Donnell
Renee Heffron
Caitlin Scoville
Kate Heller
Harald Haugen
Susan Morrison
Katherine Thomas

**World Health Organization (**WHO**)**
Ian Askew
James Kiarie
Petrus Steyn

**Eastern Cape Department of Health/University of Witwatersrand/University Fort Hare**
G Justus Hofmeyr
Mandisa Singata-Madliki

**International Centre for Reproductive Health (ICRH)/University of Nairobi**
Peter Gichangi

**Kenya Medical Research Institute (**KEMRI**)**
Nelly Mugo

**University of Zimbabwe**
Tsungai Chipato

* The ECHO Consortium Team Members dedicate this study to Dr. Ward Cates, FHI 360

CONFIDENTIAL

**Sponsor Signatory Authority**

Dr. Timothy Mastro has authority to sign the protocol on behalf of the Management Committee for all sponsoring organizations.

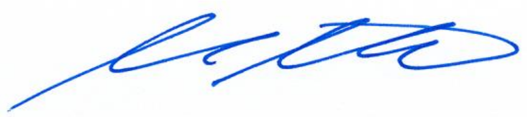

on behalf of the Management Committee __________________________________________

Dr. Timothy Mastro

CONFIDENTIAL

**A Multi Center, Open-Label, Randomised Clinical Trial Comparing HIV Incidence and Contraceptive Benefits in Women using Depot Medroxyprogesterone Acetate (DMPA), Levonorgestrel (LNG) Implant, and Copper Intrauterine Devices (IUDs)**

## INVESTIGATOR SIGNATURE FORM

**Version**

5.0

**Date**

March 3, 2017

**Funded by:**

The Bill & Melinda Gates Foundation, the U.S. Agency for International Development (USAID), the Swedish International Development Cooperation Agency (SIDA), the Medical Research Council of South Africa, and the United Nations Population Fund (UNFPA). USAID and the South African government donate contraceptives for the study.

CONFIDENTIAL

I, the Site Investigator, agree to conduct this study in full accordance with the provisions of this protocol. I will comply with the provisions of this protocol, all requirements regarding the obligations of clinical investigators as fully outlined in the International Conference on Harmonization (Section E6(R1) Good Clinical Practice), local regulatory requirements, and the Statement of Investigator, which I have also signed. I agree to maintain all study until FHI 360 advises that it is no longer necessary. Publication of the results of this study will be governed by the Consortium. Any presentation, abstract, or manuscript will be submitted to the Consortium for review prior to submission.

I have read and understand the information in the Investigator's Brochure(s), including the potential risks and side effects of the products under investigation, and will ensure that all associates, colleagues, and employees assisting in the conduct of the study are informed about the obligations incurred by their contribution to the study.

______________________________
Name of Site Investigator

______________________________ ________________________________
Signature of Site Investigator Date

**A Multi Center, Open-Label, Randomised Clinical Trial Comparing HIV Incidence and Contraceptive Benefits in Women using Depot Medroxyprogesterone Acetate (DMPA), Levonorgestrel (LNG) Implant, and Copper Intrauterine Devices (IUDs)**

## PROTOCOL SUMMARY

| | |
|---|---|
| **Short title** | The Evidence for Contraceptive options and HIV Outcomes (ECHO) Trial |
| **Design** | Multicentre, open-label, randomised clinical trial |
| **Study arms** | Random allocation to one of three study arms in a 1:1:1 ratio: depot medroxyprogesterone acetate (DMPA), levonorgestrel (LNG) implant, copper intrauterine device (IUD) |
| **Population** | Sexually active, HIV-negative women, 16-35 years old, seeking effective contraception, willing to be randomised to any of the study arms, and not desiring pregnancy for the duration of study participation |
| **Sample size** | Approximately 7,800 women (2,600 per arm) |
| **Follow-up** | 18 months per woman or until closure of the study or study site |
| **Study Sites** | Approximately 12 sites in Eastern and Southern Africa |
| **Study Duration** | Enrolment will require an estimated 18 months. Total duration of the study will be approximately 36 months from first enrolment. |
| **Operations** | The study team, in coordination with the funders and the Data and Safety Monitoring Board, will define key metrics and *a priori* criteria for stopping the trial or individual arms for operational futility as part of trial design, including % of women willing to be randomised, accrual rate, % of women discontinuing methods post-randomisation, and % loss to follow-up. |
| **Study Goal** | To answer the public health question of the relative risks (HIV acquisition) and benefits (pregnancy prevention) of three commonly-used, effective contraceptive methods among women who desire contraception |

**Primary Objective**

To compare the risks of HIV acquisition between women randomised to DMPA, levonorgestrel (LNG) implant, and copper IUDs

**Secondary Objectives**

- To compare pregnancy rates among women randomised to DMPA, LNG implant, and copper IUDs
- To compare rates of serious adverse events among women randomised to DMPA, LNG implant, and copper IUDs
- To compare rates of adverse events that lead to method discontinuation among women randomised to DMPA, LNG implant, and copper IUDs
- To compare contraceptive method continuation rates among women randomised to DMPA, LNG implant, and copper IUDs

**Tertiary Objectives**



CONFIDENTIAL

- To evaluate whether age modifies the hormonal contraception and HIV acquisition relationship
- To evaluate whether HSV-2 status modifies the hormonal contraception and HIV acquisition relationship
- To evaluate early HIV disease progression among seroconverters randomised to DMPA, LNG implant, and copper IUDs

**Primary Endpoint**

- HIV infection as measured by documented HIV seroconversion (defined by the HIV algorithm in Appendix 7) occurring post-enrolment

**Secondary Endpoints**

- Pregnancy
- serious adverse events
- Method-related adverse events resulting in method discontinuation
- Method continuation

**Tertiary Endpoints**

- HIV infection by baseline age sub-groups ≤24 years versus > 24 years
- HIV infection by baseline HSV-2 status
- HIV plasma viral load and CD4 count

CONFIDENTIAL

# 1. KEY ROLES

## 1.1 ECHO Consortium

The ECHO Consortium is committed to the One team/One study principle. The team will be convened by WHO/RHR, and the trial will be coordinated by the FHI 360/WHO led consortium. Roles have been collectively defined by our team's strengths, based on our matrix of organizations and individuals (Appendix 1).

## 1.2 ECHO Structure

A stewardship committee composed of the funders will oversee the consortium. Its main role will be assuring the financial resources and operational milestones of the trial.

A management team will provide accountability to the stewardship committee for the Consortium. It will coordinate funding among the different organizations and sites, oversee timelines for the major trial milestones, interface with the Data and Safety Monitoring Board (DSMB), plan external trial communications, and set priorities for trial publications.

The operations team is the foundational structure of the trial. It will be responsible for monitoring the daily progress of the trial. It will develop the protocol, establish site criteria, select trial sites, write the standard operating procedures (SOPs), manage trial statistics, coordinate site training, oversee site performance, and plan external trial site communications. The operations team will appoint a clinical supplies manager role with responsibility of documenting that products are manufactured, handled, and stored in accordance with applicable good manufacturing practice and in accordance with the approved protocol. A clinical supply management plan detailing the operational aspects of product management prior to product procurement will be finalized. The operations team has final responsibility for trial conduct including Good Clinical Practice (GCP) quality assurance and regulatory oversight.

The full study team will be composed of the management team, the operations team, and the site investigators of all participating sites (see Appendix 1). A directory of staff and their respective roles will be maintained in the study master file.

## 1.3 ECHO Organizational Roles

The consortium is composed of multiple organizations. Each organization will build on its organizational strengths to optimize team efficiencies. As the global normative health agency, WHO is the convening body. FHI 360 will coordinate management oversight and will be responsible for clinical monitoring, the trial master file and quality assurance audits of the trial and study sites. Wits RHI will lead on communications and good participatory practices. The University of Washington will appoint a Medical Monitor, coordinate data and clinical safety oversight and will be responsible for overall laboratory quality assurance. As the leaders in the regions where the trial evidence will have the greatest impact, country and regional experts will play key roles in Consortium operations.

## 1.4 Protocol Identification

Protocol Title: A Multi Center, Open-Label, Randomised Clinical Trial Comparing HIV Incidence and Contraceptive Benefits in Women using Depot Medroxyprogesterone Acetate (DMPA), Levonorgestrel (LNG) Implant, and Copper Intrauterine Devices (IUDs)

Clinical Trials.gov Identifier NCT02550067

Protocol Number: FHI 360 Study #523201

Short Title: The Evidence for Contraceptive options and HIV Outcomes (ECHO) Trial

Date: October 21, 2014



CONFIDENTIAL

**1.5 Sponsor Identification**

Sponsor responsibilities are shared among FHI 360, UW and WRHI, with coordination and oversight being held within the trial's Management Committee.

**1.6 Data Center**

University of Washington/ICRC
Box 359927
325 Ninth Avenue
Seattle, WA 98104

**1.7 Study Operations Coordination**

FHI 360
359 Blackwell St, Ste 200
Durham, NC 27701

## 2. INTRODUCTION

### 2.1 Background and Rationale

Hormonal forms of contraception, including progestin only injectables (depot medroxyprogesterone acetate (DMPA) and norethisterone enanthate (NET-En), are used by >150 million women worldwide [1-3]. In many settings in Africa, DMPA is the predominant contraceptive used. Women in Southern and Eastern Africa currently have limited contraceptive choices. Data from the United Nations consistently show that, in sub-Saharan Africa, when women use modern methods they are much more likely to use injectables and less likely to use long-acting, reversible methods such as the IUD when compared with women in higher income areas [4]. In most cases, this skewed method mix is due to only oral contraceptive pills and injectables being offered in contraceptive programmes [5]. The result of this is high rates of unintended pregnancy and a high level of unmet need for contraceptives. Ensuring that women have safe contraceptives available to them is of utmost importance to prevent maternal and infant morbidity and mortality.

For more than 25 years, some epidemiologic studies conducted in a variety of contexts have suggested that women using some types of hormonal contraceptives may be at increased risk of HIV acquisition; however, the results across multiple studies have been mixed, with some studies demonstrating increased HIV risk but others not showing increased HIV risk. Although initial studies on the topic often assessed all hormonal contraceptive methods together (combined hormonal contraceptives and progestin-only contraceptives with varying formulations and delivery systems), more recent studies have examined specific types of hormonal contraceptives separately [6].

The preponderance of evidence on oral contraceptive pills suggests they do not increase risk of HIV acquisition. Some studies suggest increased risk among users of injectable contraceptives, specifically DMPA . Those studies that suggest increased risk for DMPA conclude that DMPA may increase acquisition risk up to 1.5-fold [7], but it should be noted that some similar studies have failed to find such an association [8-11]. In addition, HIV acquisition risk may be higher for young women (18-24 years) compared to older women using hormonal contraception [10]. Laboratory studies have found biologic plausibility for exogenous hormones to influence HIV acquisition and pathogenesis, with DMPA having a more concerning profile than NET-En [12-14] but laboratory evidence is insufficient to provide definitive evidence of heightened HIV susceptibility as a result of progestin-only contraceptive use.

The World Health Organization (WHO) convened an expert consultation in December 2016 to review the available evidence on the risk of HIV acquisition with the use of hormonal contraception. Based on the most recent data, the WHO altered its recommendation for injectable progestin (including DMPA and NET-En) use for women at high risk of



CONFIDENTIAL

HIV from an MEC '1' (a condition for which there is no restriction for the use of the contraceptive method) to an MEC '2' (a condition where the advantages of using the method generally outweigh the theoretical or proven risks) [15]. They reiterated that women should not be denied the use of progestin-only injectables because of concerns about possible HIV risk, but should be advised about these concerns, the uncertainty about whether there is a causal relationship between progestin-only injectable use and HIV acquisition and about how to minimize their risk of acquiring HIV [15].

The WHO concluded that there continues to be evidence of a possible increased risk of acquiring HIV among progestin-only injectable users but that uncertainty exists about whether this is due to methodological issues or to a true biologic effect [15]. Based on the consistency and precision of the evidence, WHO upgraded the GRADE rating for the overall body of evidence from low to low-to-moderate for progestin-only injectable contraceptives. They also noted that even fewer data were available to assess potential HIV risk associated with the use of progestin-only implants [15]. WHO also recommended that additional high quality research evaluating the potential of increased HIV risk with injectable progestin use was a priority

An important critique of studies of the relationship between hormonal contraceptive use and HIV risk is that all have been observational; most were designed to answer a different question and the quality of data about timing and type of contraceptive method use is variable. These studies may be biased as a result of uncontrolled or unmeasured confounding factors, most prominently sexual behaviour (e.g., partner risk, condom use). Differences in behaviour can be controlled for in multivariate analyses, but only if reporting is accurate Additional observational data are unlikely to resolve this inherent limitation.

A randomised clinical trial (RCT) will provide the most definitive information concerning the comparative risk of HIV and other risks and benefits resulting from use of DMPA, LNG implant, and copper IUDs. The ultimate goal of this gold-standard study design is to provide policymakers, program managers, family planning services providers, advocates, and women conclusive information about the comparative HIV risks associated with the use of a variety of effective contraceptive methods; this information, combined with the contraceptive efficacy data for each method, will allow for better-informed family planning decision making.

**2.2 Justification for a randomised clinical trial**

Young women in parts of sub-Saharan Africa continue to have an astonishingly high incidence of HIV infection (as high as 9.0 per 100 woman-years in some studies) despite extensive prevention intervention efforts [16]. Modelling data suggest that, if a true effect, an increased risk of HIV acquisition associated with injectable progestin-only contraception [17] would have a significant impact on new infections among young women in settings with high HIV burden, predominantly in Eastern and Southern Africa.

The best way to address this uncertainty and provide clear policy guidance is with evidence from a large, well-designed, randomised clinical trial. This research design will greatly reduce many of the important limitations to the current body of observational evidence, including selection bias and many confounding factors whether known or unknown. Results from a well-conducted RCT will permit clear guidance for policymakers and programs, clearly formulated counselling messages, and ultimately allow women to make informed choices.

Failure to undertake this research will leave a critical public health question unanswered. If the data suggesting harm are true, and programs continue to offer injectable progestins in high HIV incidence settings without complete information and proper counselling and condom promotion and provision, then millions of young women face an increased risk of acquiring HIV, and the epidemic will have a powerful on-going driver. A recent model concluded that if injectable contraceptive use increased the risk of HIV infection by 1.2-2.19 fold, it could result in 27,000-130,000 new infections



CONFIDENTIAL

per year globally; 87-88% of these additional infections would occur in Southern and Eastern Africa[17]. Conversely, if false concerns about increased HIV risk persuade policymakers to discourage use or restrict provision of injectable progestins, then stopping use could cause at least 18,000 more maternal deaths per year globally, and likely even greater maternal morbidity. If the message continues to be confusing, then health care workers may stop providing injectables, even in settings with low HIV burden, and women may be scared away from an effective, relatively safe, inexpensive, widely available and accepted contraceptive method. Answering this question is critical for family planning policies, for HIV prevention, and for the health of millions of women.

### 2.3 Justification for three-arm design

A three-arm trial (DMPA, LNG implant, copper IUD) can provide the essential scientific evidence, is feasible to implement and will address the question of whether use of DMPA or the LNG implant increases the risk of HIV acquisition.

This study design provides the best estimate of the comparative risks for HIV, pregnancy, and important adverse effects for two effective progestin-only contraceptive methods and an effective non-hormonal method—the copper IUD. This trial will provide policymakers with evidence to inform contraceptive method mix and clinicians with information to counsel women about contraceptive options and HIV risk, and allow women seeking effective contraception to have the necessary information to make informed choices about potential competing risks and benefits.

The chosen methods have important biological and programmatic differences. Different progestins have different interactions with steroid (progesterone and other) receptors, which can lead to differing physiologic effects. Consequently, differences in HIV risk are possible with different progestins. This study design, in addition to evaluating the progestin injectable most implicated in HIV risk (DMPA), provides a parallel evaluation of a long-acting, low-dose progestin method (the LNG implant), along with an effective, non-hormonal contraceptive (copper IUD).

Implants are long acting and effective, and their use is rapidly increasing in areas of sub-Saharan Africa; thus, evaluation of their effects on HIV is crucial. If DMPA is found to result in higher HIV risk but the LNG implant, the implant could provide an acceptable hormonal contraceptive alternative. In addition, being able to compare each of these progestin-only methods with an effective non-hormonal method will provide robust evidence of whether use of these progestin-only methods increase the risk of HIV acquisition.

We also deliberated extensively on the merits and faults of two alternative three armed designs: DMPA, NET-En, and copper IUDs, and DMPA, NET-En, and a progestin implant (Appendix 3). However, consensus was that the advantages of the proposed arms had the optimal programmatic and scientific value. This design will provide important information for many countries where DMPA is the method of choice. Moreover, the use of progestin implants are rapidly becoming popular in East and Southern Africa and have recently been introduced in South Africa. Therefore, information on the comparative risks of the LNG implant and other methods is critical to their country program. By contrast, NET-En is less widely available elsewhere, while IUD use is growing through directed programs in some countries such as Zambia and South Africa. In addition, inclusion of the IUD as a non-hormonal comparison group may provide for improved understanding of the relationship between progestin-only contraception and HIV acquisition. Given the differences in method use in various countries, the three-arm design provides an excellent opportunity to inform women and providers about relevant contraceptive methods, while remaining feasible from an operational perspective.

We also seriously considered the value of a four-armed randomized trial that would evaluate DMPA, NET-En, a progestin implant, and copper IUDs. Although this design would allow evaluation of a different injectable progestin, the costs were prohibitive and logistical difficulties would have been increased with a four-armed trial.

CONFIDENTIAL

The inclusion of combined oral contraceptive (COC) pills, combined injectable contraceptives, hormonal IUDs, and other non-hormonal (condom) methods was also considered. However, concerns exist about including each of these potential additional arms in a clinical trial (Appendix 4). For example, a major concern for COC use is that daily adherence is variable and difficult to measure, pregnancy rates are higher, and discontinuation rates are high – potentially compromising a randomised trial if adherence or continuation is worse in one arm and also resulting in some women being assigned to a less-effective contraceptive method. A combined injectable arm has not been proposed for the final design for several reasons: these injectables i) contain estrogen, so they have more contraindications to use than progestin-only methods and may be associated with more health risks, such as venous and arterial thromboembolism, ii) are shorter acting (requiring monthly dosing), iii) have higher discontinuation rates and higher failure rates, and iv) are not widely registered in sub-Saharan Africa. Finally, a condom-only group is inappropriate because condoms are a less effective contraceptive method than the other proposed methods, their use is difficult to measure, and consistent condom use requires their partner's cooperation and thus is not a viable alternative for many women.

### 2.4 Justification of choice of comparators

This study design, in addition to evaluating the progestin injectable most implicated in HIV risk (DMPA), provides a parallel evaluation of LNG implant and a non-hormonal method (copper IUD).

#### 2.4.1 DMPA

DMPA (150 mg of medroxyprogesterone acetate (MPA)/ml) is the most commonly used injectable contraceptive worldwide, and the most commonly used method of reversible contraception in Sub-Saharan Africa. In perfect use, DMPA is highly effective and with consistent and correct use has a 0.2% failure rate; typical use failure rates are higher (~6%) [18]. Discontinuation rates can be high; one year discontinuation rates were approximately 32% in both Malawi and Kenya in DHS surveys [19], and may be particularly high among young women aged 15-19 [20], though recent HIV prevention trials have demonstrated lower discontinuation rates [21].

Advantages of DMPA include its ease of administration, its ability to be used covertly, and the fact that one injection lasts for 3 months plus a 'grace period' of 4 weeks if a woman is late for her next injection.

Although adverse effects may occur in women using this method (e.g., headache, dizziness, mood changes, acne), major problems are rare. Menstrual irregularity and spotting, along with amenorrhea, are the most common side effects [22]. While frequently cited as the main reason for early discontinuation, menstrual irregularity is considered a nuisance, and is common for all progestin-only methods, and does not present a health risk for the user. DMPA has also been associated with decreased bone mineral density (BMD) that recovers to normal after discontinuation of use [23].

#### 2.4.2 Contraceptive implants

Contraceptive progestin implants are thin rods inserted under the skin of a woman's arm. Three different implants are currently available: Jadelle and Sino-implant (II), containing levonorgestrel (LNG), and Implanon/Nexplanon/Implanon NXT, containing etonogestrel (ETG, 3-keto desogestrel). Jadelle and Sino-implant (II) consist of two silicone rods; each rod contains 75 mg of LNG. Jadelle is marketed by Bayer HealthCare, and is approved for 5 years of use; Sino-implant (II) is manufactured by Shanghai Dahua Pharmaceuticals and is labelled for 4 years of use. Implanon consists of a single rod of ethylene vinyl acetate and contains 68 mg of ETG; it is manufactured by Merck and is approved for 3 years. Nexplanon/Implanon NXT is essentially identical to Implanon, but contains 15 mg of barium sulfate, making it radiopaque, and has a pre-loaded applicator.

CONFIDENTIAL

Implants are highly effective and user independent, with failure rates of <1% for both perfect and typical use. Continuation rates for both implants average ~90% at the end of the first year and 70% by the end of the third year [24, 25].

Insertion times and ease of insertion for Jadelle and Implanon, as well as insertion-related complications, are similar [26]. Jadelle had slightly higher continuation rates than Implanon at 3 years in one study [25].

During the first year of implant use many women experience prolonged or irregular bleeding or both; in subsequent years, bleeding patterns tend to improve. Implanon users have more amenorrhea than Jadelle users over time. As with other hormonal contraceptives, headache, small weight increase, skin problems such as acne, dizziness, and mood changes have been associated with use of progestin implants. The effect of implants on BMD is marginal.

Implant use is increasing dramatically in some countries in sub-Saharan Africa; LNG and ETG implants have both been recently registered in South Africa.

For this study we have chosen to use Jadelle for several reasons:

1. Jadelle contains LNG, which is the most widely used non-MPA progestin; it is in many oral contraceptive pills including the most widely used emergency contraceptives
2. In some countries Implanon is not available in the public sector
3. LNG is the progestin used in the LNG intrauterine system, another commonly used method
4. LNG is the progestin under investigation for newer injectables
5. LNG is the progestin under investigation for use in MPT rings
6. LNG has fewer glucocorticoid effects than ETG
7. Jadelle is less hypoestrogenic than Implanon

**2.4.3 Copper IUD**

The T-380A copper IUD consists of a T-shaped polyethylene frame wound with copper wire, along with two monofilament threads to aid in removal of the IUD. IUDs may be left in place for up to 10 years. When inserted correctly, less than 1% of women using IUDs experience pregnancy in the first year, and only 2.2% of women will experience pregnancy in the first 10 years of use [27]. IUD continuation rates vary significantly and may range from 77%[28] in one year in one randomised trial in Zambia to 90% in a family planning clinic in Nigeria[29]. In Demographic and Health (DHS) surveys of non-African countries, IUD continuation rates range from 64% to 93% by one year [19].

Copper IUDs are extremely safe, effective, reversible, and forgettable, and are available worldwide, including in sub-Saharan Africa, but issues with demand, provider bias, and supplies have limited their use in most of sub-Saharan Africa. Provider concerns about IUD use, especially in young nulliparous women, include beliefs that IUDs may increase the risk of pelvic inflammatory disease (PID) and thus tubal infertility or ectopic pregnancy. IUD use in young women and those at high risk of sexually transmitted infections (STIs) has been limited in the past by such concerns. Data suggest that IUDs do not increase the risk of pelvic inflammatory disease (PID) in nulliparous women beyond a small increased risk for all women post-insertion. In a pooled analysis of the WHO clinical trials, the overall rate of PID among 22,908 IUD insertions and with 51,399 years of follow-up was 1.6/1000 woman-years (w-y) of use [30]. PID risk was highest in the first 20 days



CONFIDENTIAL

and decreased with time post IUD insertion. Women with chlamydial or gonoccocal infection at the time of IUD insertion are at an increased risk of PID relative to women without infection, but the absolute PID risk is low for both groups (0-5% for those with STIs and 0-2% for those without STIs)[31] . Additionally, IUDs do not cause tubal infertility[32], or ectopic pregnancy [33]. Even among women with HIV, PID rates with IUD insertion are low [28, 34]. IUDs are becoming more widely used in some African settings. In a recent study in Zambia, dedicated providers of long-acting methods in high volume public sector facilities inserted 11,427 copper IUDs into young women in a 14-month period [35]. IUDs are also becoming more widely used in Mozambique and Kenya.

Copper IUD users may experience heavier periods than they did before IUD insertion and may have more dysmenorrhea. However, data do not support a drop in haemoglobin as a result of Copper IUD use [36]. Current data on copper IUDs and risk of HIV infection are sparse and of low quality [1], however, they do not indicate any association between use of copper IUDs and HIV acquisition. Moreover, no other non-hormonal, highly effective, long-acting, reversible contraceptive method is available.

**2.5 Potential biological differences between different progestins**

Different progestins have different interactions with steroid receptors, which can lead to differing physiologic effects. Differential binding to the progesterone receptor (PR) leads to varying physiological effects in the vagina and uterus, ovary, breast, brain and bone. In addition to PR, the androgen receptor, mineralocorticoid receptor, and the glucocorticoid receptor (GR) also mediate the actions of some progestins. In particular, the GR is involved in regulating immune responses in humans, and could be exploited by HIV at multiple levels to ensure its pathogenic success. For example, in addition to a role in immune cells, the GR may also be involved in immune function in the cervicovaginal environment. Consequently, differences in HIV risk are theoretically possible with different progestins; such differences would most likely be due to differences in dose, metabolism, pharmacokinetics, and binding with serum proteins, as well as different affinities, specificities, and biological activities via different steroid receptors.

Evidence suggests that MPA has greater glucocorticoid activity than other progestins, a potential mechanism for a putative increase in HIV risk [14, 37]. Key differences in the clinical, biological, and immunological effects between DMPA/MPA and other progestins include:

- MPA has high affinity for the GR, which could explain its potential effect on HIV acquisition [38]
- Levonorgestrel (LNG) and norethisterone/norethindrone (NET) have lower affinity for the GR, and NET has antagonist effects, which could make these progestins less immunosuppressive than MPA[14, 38-40]
- Etonogestrel (ETG) has higher affinity for the GR than LNG and NET, less than MPA, but has still been shown to have GR mediated effects[37, 41]
- MPA has suppressive effects on reproductive tract immune markers, while preliminary data suggest NET-En does not exert immunosuppressive activity at concentrations up to 100 times higher than DMPA[42-45]
- Intramuscular (IM) DMPA is high dose, and leads to high progestin levels compared with implants. IM DMPA leads to hypoestrogenism with profoundly low estradiol levels (15-50 pg/ml), which has been hypothesised to be another mechanism by which progestin contraceptives might increase HIV risk
- DMPA often leads to amenorrhea, while LNG users have less amenorrhea than DMPA users.

**2.6 Contraceptive Continuation**

Poor or differential continuation of allocated contraceptive methods could undermine the randomised design and compromise study validity. The study team has anticipated the importance of users continuing with their assigned



CONFIDENTIAL

contraceptive method. Historically, contraceptive method continuation rates for the three methods proposed in this study have ranged from 50 to 90% at 12 months depending on the study setting and population.

Few randomised trials have assigned women to different contraceptive methods and also evaluated contraceptive continuation rates among these women. In one study researchers enrolled 368 women in Egypt, Brazil, Guatemala, and Vietnam. At 12 months, 80% of women allocated to DMPA and 85% women allocated to the IUD were still using their assigned method [46]. Another small RCT compared the LNG IUD with Jadelle implants in China [47]. In this study of 200 women, 12-month continuation rates were 90% and 96%, respectively. In Zambia, 599 HIV-infected post-partum women were randomised to copper IUDs or hormonal contraception[28]. In this trial, 69% of women continued their assigned contraceptive method over 24 months (87% of hormonal contraceptive users and 51% of IUD users). However, median time to discontinuation was 475 days for the DMPA group and 408 days for the IUD group. Thus, through 12 months, about 95% of hormonal contraceptive users and 77% of IUD users continued using their methods. Another recent trial randomized 200 HIV infected women in Malawi to DMPA or copper IUD[48]. In this study, 68% of Cu-IUD and 65% of DMPA users continued their methods through 48 weeks; partner disapproval, heavy bleeding, and no previous use of contraception were associated with risk of discontinuation. Finally, a recent secondary analysis of 262 South African adolescents (ages 15-19, median age 18 years) enrolled in a study of bone mass density reported that the 1-year continuation rates for DMPA was 40.4% [20].

Fears and misbeliefs contribute to method discontinuation in many women requesting IUD removal [49], and structured and extensive counselling may help mitigate these fears [50]. In one randomised trial in Mexico, women who received pre-treatment, structured information about DMPA were more likely to continue the method than were women who received routine counselling[51]. Recent studies have found that method continuation can be improved with careful planning and counselling. In the largest cohort study of hormonal contraception and HIV study to date, contraceptive continuation rates at 1 year were 78% for DMPA users [52]. In a recent large US cohort study, high continuation rates were seen among young women beginning long-acting reversible contraceptive methods including IUDs and implants, with use of DMPA, IUDs, and implants also associated with very low failure rates (<0.3 pregnancies per 100 person-years)[53, 54]. In this study, designed to focus on improving use of long-acting methods, 12-month continuation rates were: LNG intrauterine system (88%), copper IUD (84%), implant (83%), and DMPA (57%)[54]. Lastly, among women who were not using effective contraception prior to enrolling in the recent FEM-PrEP HIV trial but who chose to use an injectable method (DMPA or NET-En) to fulfil the study's contraceptive use requirement, 82% continued to use their injectable for an average of 9 months of study participation, despite switching to other methods being permitted per protocol (88% of existing injectable users so continued)[55]. In summary, these studies demonstrate that in structured research settings in sub-Saharan Africa continuation rates for DMPA can range from 40 to 85%, and continuation rates for implants and copper IUD can range from 68% to 96%.

In the proposed ECHO trial, potential study participants will be carefully assessed and counselled prior to randomisation, to ensure that they are willing to be randomised, willing to defer pregnancy and to use their assigned method for their duration of study participation. The length of study follow-up (18 months or until closure of the study or a study site) was chosen because women commonly use longer acting contraceptives for at least this length of time, and it would ensure the required number of participants while also not placing undue burdens on either women or the sites. Intensive, on-going counselling will be provided to study participants about the side effects associated with each of the three contraceptive methods, because method discontinuation is most often due to side effects, especially those that are unanticipated. Finally, each participant will be scheduled for a follow- up visit at one month after her randomisation to focus on early side effects/tolerability to assist with method start-up symptoms that often abate. Obtaining the best

CONFIDENTIAL

possible contraceptive continuation rates will be addressed through following 'best practices' strategies reported in previous studies as well as linkages with family planning programs to help women transition after the study.

## 3. OBJECTIVES AND ENDPOINTS

### 3.1 Primary Objective
To compare the risks of HIV acquisition between women randomised to DMPA, LNG implant, and copper IUDs

### 3.2 Secondary Objectives
- To compare pregnancy rates among women randomised to DMPA, LNG implant, and copper IUDs
- To compare rates of serious adverse events among women randomised to DMPA, LNG implant, and copper IUDs
- To compare rates of adverse events that lead to method discontinuation among women randomised to DMPA, LNG implant, and copper IUDs
- To compare contraceptive method continuation rates among women randomised to DMPA, LNG implant, and copper IUDs

### 3.3 Tertiary Objectives
- To evaluate whether age modifies the hormonal contraception and HIV acquisition relationship
- To evaluate whether HSV-2 status modifies the hormonal contraception and HIV acquisition relationship
- To evaluate early HIV disease progression among seroconverters randomised to DMPA, LNG implant, and copper IUDs

### 3.4 Primary Endpoint
- HIV infection as measured by documented HIV seroconversion (defined by the HIV algorithm in Appendix 7) occurring post-enrolment.

### 3.5 Secondary Endpoints
- Pregnancy
- Serious adverse events
- Adverse events resulting in method discontinuation
- Method continuation

### 3.6 Tertiary Endpoints
- HIV plasma viral load and CD4 count
- HIV infection by baseline age sub-groups ≤24 years versus >24 years
- HIV infection by baseline HSV-2 status

## 4. STUDY DESIGN

### 4.1 Trial Design
This is a multi-centre, open-label, randomised clinical trial designed to compare the risks of HIV acquisition between women randomized to one of three commonly used, effective contraceptive methods. Approximately 7,800 HIV antibody negative women aged 16-35 years will be enrolled and randomised in a 1:1:1 ratio to DMPA, LNG implant or



CONFIDENTIAL

the copper IUD and followed for 18 months of contraceptive method use. The study size and follow-up is expected to yield at least 250 HIV endpoints for each of the three method comparisons: copper IUD vs. DMPA, LNG implant vs. DMPA, copper IUD vs. LNG implant, yielding 80% power to detect 50% increases (equivalently, 33% reductions) in risk of HIV between any two methods. Enrolment is expected to take approximately 18 months to complete, and the total duration of the study in the field will be approximately 36 months from first enrolment.

**4.2 Study Setting**

The Southern and Eastern Africa region remains the area most heavily affected by the HIV epidemic, with 34% of persons living with HIV/AIDS residing in 10 countries in Southern Africa [56]. The study will therefore target approximately 11 sites with high incidence (target average ≥3.5/100w-y) in eastern and southern Africa, each enrolling approximately 400-800 participants.

Study sites under consideration include sites in South Africa, Kenya, Zambia, and Swaziland. The ECHO team has asked potential sites in these countries to complete surveys describing site capacity, using pre-determined metrics (Appendix 5). Key criteria for site selection include estimated HIV incidence, research capacity, contraceptive experience, investigator availability, appropriate population for the trial, and available clinical and laboratory infrastructure. The operations team will be responsible for site selection.

**Table 1: Contraceptive options in proposed countries by registration status and public sector availability**

| | DMPA | | LNG Implant | | Copper-IUD | |
|---|---|---|---|---|---|---|
| | **Registered** | **Available** | **Registered** | **Available** | **Registered** | **Available** |
| Kenya | X | X | X | X | X | X |
| South Africa | X | X | X | X | X | X |
| Swaziland | X | X | X | X | X | X |
| Zambia | X | X | X | X | X | X |

**4.2.1 HIV prevalence and incidence in the proposed countries**

Although the incidence of new HIV infections in Eastern and Southern Africa has decreased when compared with ten years ago [57], prevalence remains high throughout the region [58]. HIV incidence data in young women from a variety of African settings are available from recently published HIV prevention trials. In the FEM-PrEP and VOICE trials, assessing the effectiveness of pre-exposure prophylaxis for HIV prevention, the HIV incidence rates in the placebo group were approximately 5 per 100 woman-years [59] and were >6 per 100 woman-years for some sites in South Africa.

Although estimates of HIV incidence by age group are difficult to ascertain, modelling studies for South Africa suggest that the highest incidence among women occurs between age 15-19 (estimated at 2-3% per year) and 20-24 (estimated at 3-4% per year), with slow but steady declines in incidence starting from age 25 (estimated at between 1.5-2.5% per year at age 25-29, between 1-2.5% at age 30-34, between 0.5-2% at age 35-39, and between 0.5-1.5% at age 40-44) [60]. In order to focus on enrolling women at the highest risk of acquiring HIV, this trial will enrol women aged 16 to 35 years.

**4.3 Study Population**

The study will enrol sexually active, HIV-negative women, 16-35 years old, seeking effective contraception, willing to be randomised to any of the study arms and not desiring pregnancy for the duration of study participation. Women will be recruited from family planning/reproductive health clinics, clinics serving post-partum and post-abortion clients, other relevant clinics, and the general community.



CONFIDENTIAL

## 4.4 Eligibility Criteria

### 4.4.1 Inclusion Criteria

To be eligible for the study a woman must meet all of the following criteria:

- 16-35 years of age
  - Previously pregnant 16 and 17 year olds, where permissible by national regulations and local IRB approval
- HIV-seronegative
- Wants to use effective contraception
- Is able and willing to provide written informed consent
- Agrees to be randomised to either DMPA, LNG implant, or copper IUD
- Agrees to use assigned method for 18 months
- Agrees to follow all study requirements
- Intends to stay in the study area for the next 18 months, and willing and able to provide adequate locator information
- If has had a recent third trimester birth, is at least 6 weeks postpartum at time of enrolment
- Is sexually active (has had vaginal sex within the last 3 months) or was pregnant within the last 3 months
- Agrees not to participate in studies of drugs or vaccines or any other clinical research study while participating in this study.

### 4.4.2 Exclusion Criteria

A woman who reports or is found to have any of the following criteria will be excluded from the study:

- Medical contraindications (Category 3 or 4 criteria as detailed in the WHO MEC[1] to DMPA, LNG implant, or copper IUDs, including
  - Persistent measured blood pressure 160/100 mmHg or higher, or history of uncontrolled hypertension due to vascular disease
  - History of a stroke or heart attack
  - History of rheumatic disease such as lupus, with positive or unknown antiphospholipid antibodies or low platelet count
  - Multiple risk factors for cardiovascular disease including two or more of: a) smoked regularly for the past 6 months, b) history of hypertension, c) history of diabetes, and d) history of abnormal cholesterol/lipid disorder

[1] World Health Organization (WHO), *Medical eligibility criteria for contraceptive use*. 5th ed 2015, Geneva: WHO



CONFIDENTIAL

- Diabetes for more than 20 years or diabetes with nephropathy, retinopathy, or neuropathy
- A current/acute blood clot in her legs or lungs
- History of severe liver cirrhosis or a liver tumor
- Current or previous breast cancer
- Endometrial, ovarian, or cervical cancer
- History of trophoblastic disease
- Unexplained vaginal bleeding between menstrual periods or bleeding after intercourse
- History of pelvic tuberculosis
- Anatomical abnormality of the uterus incompatible with IUD insertion[2]
- A recent septic abortion
- Untreated mucopurulent cervicitis on exam, untreated pelvic inflammatory disease (PID), or untreated known gonorrhoea or chlamydia[3]

- Has received a DMPA or NET-En injection in the last 6 months
- Has used an implant or an IUD in the last 6 months
- Is pregnant or intending to become pregnant in the next 18 months
- Has had a hysterectomy or sterilization
- Has previously enroled in the study
- Has any condition (social or medical), which in the opinion of the investigator, would make study participation unsafe or complicate data interpretation.

**4.4.3 Inclusion of Adolescents 16-17 Years Old**

Young women 16 and 17 years of age who have previously been pregnant and where permissible by national regulations and local IRB approval will be invited to participate. The inclusion of this population is important for several reasons. In many countries in Sub-Saharan Africa women 16-17 years of age have high rates of HIV incidence and also need effective contraceptive options. Thus, we anticipate in many countries this population will make up an important part of the study population and the HIV outcomes. Moreover, as the study research question is of particular importance to this group and to make findings generalisable, they should form part of the study population. Finally, there is a possibility that the effect of some hormonal contraceptives on HIV risk is greater among younger (rather than older) women and this underscores the importance of their inclusion.

**4.5 Co-enrolment Criteria**

As indicated in Section 4.4, participants should not take part in other research studies involving drugs, devices, vaccines, or other clinical research while taking part in this study. Each site will be responsible for defining procedures for

[2] This will not be known until enrolment, when in a woman who is randomized to IUD, difficulty is noted passing a uterine sound

[3] May be enroled after treatment



CONFIDENTIAL

management and prevention of co-enrolment prior to site initiation. Participation in studies not involving biomedical interventions will be permitted.

Exceptions to this co-enrolment prohibition may be made for the following types of studies, at the discretion of the site investigator /designee:

- Ancillary studies developed and approved by the ECHO management team
- Interventional studies for persons with HIV infection for those women who seroconvert during the trial

Should any participant report or should study staff discover concurrent participation in contraindicated studies after randomisation, the site investigator/designee will consult the ECHO operations team regarding on-going product use and other potential safety considerations associated with co-enrolment.

**4.6 Interventions**

Study procedures including provision of contraceptives will begin only after the study has been approved by participating IRBs and applicable regulatory authorities for each of the participating sites.

**4.6.1 Description/ Regimen of Study products**

Sites will randomise each enrolled participant to one of three study interventions: DMPA, LNG implant or the copper T380-A IUD. Product manufacturing, labelling, and management information will be documented in a Clinical Supplies Management Plan.

**4.6.2 Administration/insertion of study products**

*DMPA administration*

For women randomised to DMPA, site staff will inject the initial IM injection of DMPA (medroxyprogesterone acetate sterile aqueous suspension 150 mg per 1 mL) at enrolment. Subsequent injections will be given every 3 months (i.e., at quarterly study visits) at the study site.

Sites will assess participants who are more than 4 weeks late for their DMPA injections for pregnancy prior to administering a subsequent injection.

*LNG implant insertion*

Women randomised to implants will receive LNG implants at enrolment. Trained clinicians will insert the implants under the skin of a woman's inner side of the upper arm using standard manufacturer recommended techniques. Sites will observe women for 10 minutes after implant insertion. Women will be shown how to palpate for the rods.

*T380a Copper IUD insertion*

Women randomised to IUDs will receive their IUDs at enrolment. Trained providers will insert T380a copper IUDs using standard insertion techniques. During the screening visit, all participants will be screened for gonorrhoea and chlamydia. Participants do not need to have available gonorrhoea and chlamydia testing results to receive their IUDs, and scheduling of the enrolment visit <u>should not</u> be delayed to wait for results. If test results return before enrolment and are positive the participant should be called back and treated appropriately, and the IUD may be inserted immediately. If a participant has clinical signs or symptoms of PID, or mucopurulent cervical discharge at time of the enrolment visit, treatment will be provided and IUD insertion will be delayed by at least 7 days. For a few asymptomatic women, IUD insertion might occur before a positive screening test result becomes available. In these participants, treatment will commence as soon as possible once results of a positive test are received. IUDs do not need to be removed. This will not



CONFIDENTIAL

impact participant safety; treatment of PID after IUD insertion, as well as same-day insertion protocols have been shown to be effective and safe [61-63].

#### 4.6.3 Supply and accountability

Study sites will start administering contraceptive products only after the study has been approved by participating IRBs and applicable regulatory authorities. Each study site will order contraceptives on a timely basis to ensure a sufficient supply throughout the study.

Sites will store study contraceptives according to the labelled storage requirements for each product in a limited access securely locked area. For purposes of inventory accountability, the study site will make supplies of hormonal contraceptives obtained for the study available only to study staff and these contraceptives will be provided only to women enrolled in the study.

#### 4.6.4 Strategies for successful contraceptive initiation and assessing continuation

*Ensuring successful insertion of IUDs and implants*

Physician education and training regarding the few contraindications to, and the low risks of, inserting IUDs in young and nulliparous women will be conducted prior to study initiation with refresher trainings as needed. In addition, sites will screen all women for chlamydia and gonorrhoea and treat those with positive results.

Experienced trainers will train site clinical personnel in standardised IUD and implant insertion before the study begins. Only providers trained and skilled in insertion and removal of implants and IUDs will do insertions and/or removals during the study.

*Assessing method continuation*

The study team will assess contraceptive method use by study records and participant self-report. Sites will request that women receive their DMPA injections at the study sites, and administered injections will be recorded directly. If a participant reports getting an injection elsewhere, she will be asked to show her contraceptive record (if available); otherwise she will be asked to recall to the best of her ability the date of her last injection. Sites will ask women using implants or IUDs about continued use at each regular visit. For women in the implant arm, implant presence will be verified by palpation at each visit. At the final study visit, study clinicians will validate presence of the implant by palpation of the implant site and presence of the IUD by visualization of IUD strings. Inability to verify the presence of either the IUD or the implant will lead to radiologic tests for confirmation.

*Improving method continuation*

In general, continuation rates for contraceptive methods, particularly progestin-only injectables, are lower in typical-use settings than is desired for this trial. Before randomisation, trained study staff will counsel women extensively on the importance of using their assigned methods for their full study participation. The local communities will be engaged to defray contraceptive myths and misperceptions and potential obstacles to method initiation or continuation in the target population. Strategies for promoting method continuation and retention will be developed that engage study site leaders, study site staff, current contraceptive users, and the family planning clinic community. All participants will return at one month to assess any early problems with their methods. Trained staff will repeat structured counselling at each scheduled study follow-up visit to maximise method continuation. Furthermore, study staff will ask any study participant who is considering discontinuing her method to come discuss this before doing so, so that the study staff can help with any issues about method side effects and to dispel any myths. By advising women ahead of time about potential side effects of the method and how to manage them, as well as having a contraceptive "trouble-shooting" visit



CONFIDENTIAL

one month after enrolment and ensuring availability of study staff for contraceptive counselling at any time the study team anticipates that method continuation will be higher than what has been reported in standard practice (Section 2.6).

#### 4.6.5 Contraception discontinuation

Women who discontinue their contraceptive method will be asked to continue with study visits as scheduled. Potential reasons women may discontinue their study product include:

- *Participant desire:* A participant may discontinue her allocated method of contraception at any time if she wishes, for any reason. However, women will be asked to visit the study site prior to discontinuation to discuss their reasons for switching. If their reasons for discontinuation are due to side effects that can be anticipated to improve or can be managed, women will be counselled on ways to manage the side effects and advised that they are likely to get better. If after this counselling they still wish to discontinue, they will be offered another method of their choice, referred if their preferred method is not available at the study site or given the option to use no contraceptive.
- *Allergic reaction to study product:* Although allergic reactions to the study products are rare, some may occur. Mild allergic reactions will be managed conservatively. Very rarely, severe or persistent allergic reactions could lead to discontinuation of the study method, either based on clinician judgment or participant desire. Sites will offer any participant who discontinues her method due to an allergic reaction another contraceptive method of her choice.
- *Pregnancy*: Women who become pregnant will be managed as in Section 5.4.9. Women who become pregnant with the IUD *in situ* will have a pelvic exam when the pregnancy is identified and the IUD will be removed at that time if possible or as soon as possible according to standards of care. Women who become pregnant with the implant *in situ* will be managed depending on their pregnancy intentions and within the context of local laws. Women who become pregnant while using DMPA will not get their next scheduled dose of DMPA unless there is resolution of the pregnancy in the intervening weeks. Following resolution of their pregnancy, women will be provided their allocated study method, if still in the study period.
- *Safety*: Women who develop any medical condition for which continuation of their allocated method is considered a contra-indication (for instance, a woman allocated to DMPA who develops breast cancer) will have their method discontinued. Site investigators may also discontinue study products for other immediate safety concerns as needed.

#### 4.6.6 Concomitant Medications

Women on liver enzyme-inducing medications (Appendix 10) are not excluded from the study, however use of such medications will be documented. Sites will counsel women of the potential increased risk of pregnancy, and may recommend additional barrier protection. Other concomitant medications will not be documented.

## 5. STUDY PROCEDURES

### 5.1 Study Coordination

The ECHO operations team will develop a common manual of operations (MOP) to guide implementation; this manual will provide instructions and guidance on study visits and procedures; data and forms processing; specimen collection, processing, storing, and shipping; AE – including SAE - assessment, management and reporting; dispensing study products and documenting product accountability; and other study operations. The team will also provide standardised study-specific training to all sites before site initiation.

CONFIDENTIAL

Close coordination between study team members is necessary to track study progress, respond to queries about proper study implementation, and address other issues in a timely manner. The operations team will address issues related to study eligibility and AE management and reporting as needed to assure consistent case management, documentation, and information sharing across sites. The team will also monitor rates of accrual, contraceptive continuation, follow-up, and AE incidence.

**Clinical Settings**

Recruitment and pre-screening will occur in various sites, as outlined below (section 5.2). Screening, enrolment, and all study procedures, including clinical exams, data collection, and point of care laboratory testing, will occur at study sites, which may be clinical sites that have research capacity (with dedicated space for research), or separate free-standing research units (See Appendix 5 for site selection criteria). Chosen sites will participate in training prior to study initiation to ensure high quality contraceptive provision. Standard operating procedures on contraceptive provision will be developed. Every site will additionally submit a site-specific protocol for review.

### 5.2 Recruitment/Pre-Screening

Sites will recruit participants from a variety of sources, including family planning clinics, post-partum and post-abortion clinics, primary care clinics, and the general community. Participants may be referred to the study from other local research projects and other health and social service providers serving the target study population. Each site will establish local recruitment methods that operationalize protocol-specified requirements (including target HIV incidence) and ensure participant privacy for eligibility determination in a manner that is tailored to and most efficient for the local study setting. Recruitment materials will be approved by site IRBs/ECs prior to study initiation. Each site will undertake activities such as posters and general information sessions to inform potential participants.

Trained research staff will counsel potentially eligible women about the available contraceptive methods and about possible participation in the trial. They will inform women about the trial including the reasons for conducting the trial. Women committed to using effective contraception for at least 18 months who indicate a willingness to be randomised to any of the three contraceptive methods and to undergo HIV testing will be referred to the study site for a screening visit.

### 5.3 Study visit schedule

During the screening visit, staff will review the general schedule of participation with the potential participant. Women will be asked to participate in the study for 18 months unless their study site is closed before the 18 months is completed. After being enrolled, participants will return at one month to specifically review contraceptive complications and receive contraceptive counselling, then quarterly thereafter.

### 5.4 Clinical Evaluations, Procedures and Management

#### 5.4.1 Medical, contraceptive, and reproductive history

At the screening visit, study staff will ask women about their medical history, including specific questions about any medical problems or current medications that are contraindications to the use of any of the study methods (Section 4.6.6, Appendix 9). Women will also be asked about their past use of contraceptive methods as relevant and details about their previous pregnancies, if any.

CONFIDENTIAL

### 5.4.2 Physical exam

Trained clinicians will do brief physical exams on all potential participants at screening to evaluate for contraindications to the study methods. These exams will include measurement of blood pressure, height, and weight, and pelvic exams. At the time of the pelvic exam an endocervical swab will be taken to test for *Neisseria gonorrhoeae* and *Chlamydia trachomatis*. A bimanual exam will be performed to assess for the presence of any uterine or cervical anomalies that would preclude IUD use (see Section 4.4.2). Any suspected reproductive tract infections identified on physical exam will be treated syndromically, according to local standards of care. In addition, treatment will be initiated and/or adjusted as appropriate based on lab results when available.

### 5.4.3 Laboratory evaluations

Required laboratory evaluations (Appendices 5 & 6) include (see Visit Procedures sections 5.5-5.8):

- HIV rapid testing (blood)
- HIV confirmatory testing (blood)
- HIV RNA quantification (blood)
- CD4 T-cell count (blood)
- HSV-2 serology
- Pregnancy testing (urine)
- Nucleic acid amplification testing for *Neisseria gonorrhoeae* and *Chlamydia trachomatis* (endocervical swab)
- Plasma archival, serum archival
- Endocervical archival

The intention of the study team is that protocol-defined laboratory evaluations will be conducted in laboratories associated with each study site. However, shipment of samples, for quality assurance or for specialized testing not available at the site laboratory, may be required. Shipment of samples will be done only with the approval of the local ethics review committee and under other local regulatory guidance.

### 5.4.4 Discontinuation of study product in response to observed adverse events

All three study interventions have been in wide use for many years, and unexpected related adverse events as may be encountered in the study of new investigational products are extremely unlikely. Any related events, if serious, may be considered a reason for discontinuation of the allocated method, with the participant's well-being the primary consideration. The site clinicians, in consultation with the core safety team, will make these decisions. Such events might include, but are not limited to, deep venous thrombosis or pelvic infection unresponsive to treatment.

Known non-serious adverse events associated with progestin-only contraceptive methods (mainly menstrual cycle disruption) will be treated conservatively (e.g., NSAIDs), and will be a reason for discontinuation only if requested by the participant, and after counselling about the need for on-going contraception, pregnancy risk and advantages and disadvantages of alternative methods.

Similarly, common side-effects of the IUD (mainly menorrhagia and dysmenorrhea) will be managed as in routine clinical practice, with offer of conservative management (e.g. NSAIDs or tranexamic acid) and discontinuation on request of the participant after counselling as above.

CONFIDENTIAL

### 5.4.5 Sexually transmitted and reproductive tract infections and other clinical findings

Although such tests are not required for DMPA, LNG implant or IUD insertion, all women will be tested for gonorrhoea and chlamydia at screening. These infections are important potential cofactors in HIV acquisition and therefore must be assessed at study entry in all participants.

Pelvic infection is expected to be rare (1/600 in a randomised trial of hormonal contraception versus the IUD in HIV infected women at high risk in Zambia)[28], and any such occurrence after the post-insertion period is unlikely to be related to the IUD. If PID occurs after IUD placement, women who retain their IUDs during and after treatment for the infection have similar or improved outcomes compared with those who have their IUDs removed[62].

In this study, the study team will follow WHO guidelines. WHO guidelines do not recommend the use of routine antibiotic prophylaxis prior to IUD insertion [64]. Although South African guidelines currently do recommend their use due to high prevalence of STIs, this recommendation is in the setting of lack of routine access to STI screening tests. In this study, all women will be screened for STIs, and as a result prophylactic antibiotics are not necessary.

A woman with an IUD diagnosed with PID will be treated without removal of the IUD [61, 64], unless she wishes to discontinue the method after counselling. Clinical improvement will be monitored closely. If the infection does not improve with adequate antibiotic therapy, the IUD will be removed. Other infections or clinical events will be managed in accordance with routine service.

Testing for gonorrhoea and chlamydia will also occur at the final visit (which may be the seroconversion visit) in order to get information on their final STI status and to ensure women are gonorrhoea/ chlamydia free at the end of the study. Final STI status will inform sensitivity analyses and any women with positive test results will be called back for treatment, if necessary.

Women will be offered the option for partner treatment, according to local regulations. Contact tracing and notification will not be performed, unless required by local regulations. This will be further specified in site-specific protocols.

### 5.4.6 Cervical Cancer Screening

Sites may screen appropriate participants for cervical cancer at the initial screening pelvic exam according to local standard of care and where appropriate diagnosis, follow-up, and treatment are available. Screening methods may include visual inspection with acetic acid (VIA), cytology where cytopathology services are available, or other methods.

### 5.4.7 Communicable Disease Reporting

Study staff will comply with local requirements to report communicable diseases including HIV identified among study participants. Participants will be made aware of reporting requirements during the informed consent process.

### 5.4.8 HIV infection

Sites will fully counsel participants who seroconvert and refer them to local HIV care providers for on-going care, including CD4 cell count and antiretroviral treatment according to local policy. Seroconverters will be asked to remain in the study until completion of the follow-up period in order to continue data collection relevant to secondary outcomes.

### 5.4.9 Pregnancy

Study staff will offer or refer women who become pregnant options for further care, according to their wishes and in keeping with local laws. If the pregnancy continues, women will discontinue their assigned method but will remain in the study to the end of the follow-up period in order to continue data collection relevant to the primary and other

CONFIDENTIAL

secondary outcomes. Should the pregnancy terminate prior to completion of study follow up, women will be encouraged to continue or resume their allocated method of contraception, but will be offered their choice of any study method. If the pregnancy continues beyond the end of the study, women will be referred for further care.

**5.4.10 Criteria for early discontinuation of study participation**

Participants who decline to remain in the study will be discontinued. Stopping or changing the allocated method will not be a reason for study discontinuation. Pregnancy or HIV seroconversion will not be a reason for discontinuation, as other outcomes will remain relevant.

**5.4.11 Management of discontinuation/changing of contraceptive method**

Women who request a contraceptive method change will first be counselled about concerns with their allocated method and, if appropriate, will be encouraged to continue the allocated method. Women who, after counselling, still wish to change contraceptive methods will be offered the study-provided method of their choice or referred for alternative methods and will remain in the study to the end of the follow-up period in order to continue data collection relevant to the primary and other secondary outcomes.

**5.4.11.1 DMPA**

If a participant wishes to discontinue DMPA, the next scheduled injection will be omitted, and alternative contraception offered.

**5.4.11.2 IUD or Implant**

The IUD or implant will be removed at any time requested by the participant. An alternative method will be offered. All sites will have trained clinicians available for IUD or implant removal, either on-site or within immediate referral.

**5.5 Screening visit**

At the screening visit study staff will conduct administrative and regulatory procedures (including obtaining written informed consent and/or parental consent with minor assent for women under the age of legal consent), provide contraceptive and HIV counselling, do pelvic examinations, obtain reproductive health data and test for HIV using rapid tests (Table 2 & Appendix 6). Potential participants will provide written consent before any screening procedures are initiated. Women who are found not to be eligible for the study will be referred for further care, if the services they require are not available at the research site or clinic. Similarly, women who are found to be HIV positive at the screening visit will be referred for HIV care. These referral linkages will be established prior to study initiation, if they do not already exist.

**Table 2: Screening Visit Procedures**

| Component | Procedures |
|---|---|
| **Administrative and Regulatory** | • Obtain written informed consent<br>• Assign Participant Identification number<br>• Assess eligibility<br>• Collect locator information<br>• Provide reimbursement<br>• Schedule next visit |

CONFIDENTIAL

| | | |
|---|---|---|
| **Behavioural/Counselling** | | • Contraceptive counselling, including contraceptive continuation on all study methods<br>• HIV/STI risk reduction, including condom use and provision<br>• HIV pre- and post-test counselling |
| **Clinical** | | • Collect demographic information<br>• Obtain medical, contraceptive, reproductive history/data<br>• Measure height, weight, and blood pressure<br>• Conduct pelvic examination<br>• Screen for cervical cancer (optional –based on local standards of care and availability of treatment)<br>• Assess for risk of pregnancy<br>• Conduct syndromic assessment/treatment for STIs/RTIs |
| **Laboratory** | Urine | • Test urine for pregnancy as necessary |
| | Pelvic | • Collect endocervical specimens for Nucleic Acid Amplification Test (NAAT) for *Neisseria gonorrhoeae* and *Chlamydia trachomatis* (GC/CT)<br>• Endocervical swab (archival) |
| | Blood | • Test blood for HIV (rapid test) |

CONFIDENTIAL

**5.6 Enrolment visit**

Sites will schedule women for enrolment visits within a minimum of 1 day to a maximum of 6 weeks (42 days) of screening. To ensure that women requesting contraception are not delayed sites will attempt to have most women return within 1 week for enrolment. If a potential participant returns for enrolment beyond 42 days, she may be rescreened and then enrolled on the same day. At enrolment, sites will review study eligibility, and obtain enrolment informed consent or parental consent and assent from minor participants for women under the age of legal consent (for sites which opt to use separate screening and enrolment consents only). Study staff will collect limited medical, reproductive, and behavioural data. Women will provide urine for pregnancy testing and blood for plasma archiving. Sites will randomise eligible women (at which point a woman will be considered enroled) and women will receive one of the three study contraceptive methods. Women will also be scheduled for their 1-month contraceptive follow-up visit (Table 3 & Appendix 6).

**Table 3: Enrolment Visit Procedures**

| **Component** | **Procedures** |
|---|---|

| | | |
|---|---|---|
| **Administrative and Regulatory** | | • Obtain written informed consent for enrolment (for sites which opt to use separate screening and enrolment consents only)<br>• Review and confirm eligibility<br>• Review/update locator information<br>• Obtain random allocation<br>• Provide reimbursement<br>• Schedule next study visit |
| **Behavioural/Counselling** | | • Assess sexual and reproductive risk behaviour including sexual practices, condom use), contraceptive use, etc.<br>• Provide counselling:<br>− method related side effects and continuation<br>− HIV/STI risk reduction including condom use<br>− Protocol adherence |
| **Clinical** | | • Obtain/update medical, contraceptive, reproductive history/data<br>• Administer assigned contraceptive method (which will include a pelvic exam for women randomized to IUD)<br>• Conduct syndromic assessment/treatment for STIs/RTIs |
| **Laboratory** | Urine | • Test urine for pregnancy[1] |
| | Blood | • Collect blood for plasma/serum archive<br>• Collect blood for HSV-2 testing |

[1]If a woman has had a clinician verified (by medical record review, knowledge of completed evacuation of uterus, observation of the expelled products of conception, or ultrasound confirmation) miscarriage or pregnancy termination within 12 days, a urine pregnancy test is not required. If such a woman has a pregnancy test done which is positive, she may still be enroled.

## 5.7 Follow-up visits

### 5.7.1 One-month follow-up visit

At 1-month all women will have a follow-up visit to receive further counselling on their contraceptive method and to discuss any potential concerns or contraceptive side effects. Study clinicians will also do pelvic examinations on women in the IUD arm, to verify the presence of the IUD strings, and will verify implant presence by palpation for women in the implant arm. Inability to verify the presence of either IUD or implant will lead to radiologic tests for confirmation.

### 5.7.2 Quarterly (3-monthly) follow-up visits

Participants will return for scheduled follow-up visits at 3, 6, 9, 12, and 15 months. Visits will consist of: contraceptive and HIV counselling, limited behavioural assessment, review of relevant adverse events, syndromic assessment of STI/RTIs, assessment for pregnancy, and provision of injectable contraception (Table 4 & Appendix 6). Blood will be collected for HIV rapid testing (all visits) and for plasma archiving (6 months only). For women testing positive for HIV, refer to section 5.10. Women will be scheduled for their next follow-up visit.

CONFIDENTIAL

**Table 4: Follow-up Visit Procedures**

| Component | | Procedures |
|---|---|---|
| **Administrative and Regulatory** | | • Review/update locator information<br>• Provide reimbursement<br>• Schedule next study visit |
| **Behavioural/Counselling** | | • Assess sexual risk behaviour including sexual practices, condom use, etc.<br>• Provide counselling:<br>− method related side effects and continuation<br>− HIV/STI risk reduction including condom use<br>• Protocol adherence |
| **Clinical** | | • Assess for adverse events (section 9)<br>• Collect contraceptive use information<br>• Provide contraceptive injections, as indicated<br>• Conduct syndromic assessment/treatment for STIs/RTIs)<br>• Assess risk of pregnancy |
| **Laboratory** | Urine | • Test urine for pregnancy as necessary |
| | Blood | • Test blood for HIV (rapid test)<br>• Collect specimen for archiving (6 month visit only) |

**5.7.3 Final study visit**

At 18 months or at any time a participant discontinues the study, she will have a final study visit. This visit is similar to other follow-up visits with the addition of pelvic exams, endocervical swabs for testing for gonococcal and chlamydial infection and archiving, urine hCG, and blood for plasma archiving (Table 5 & Appendix 6). Women testing positive for chlamydial or gonococcal infections will be recalled to the study clinic for treatment. Women will be re-counselled about their contraceptive choices. Any women who wishes can have their study assigned method removed (IUD or implant) and can have any new study method provided if she wishes. All study participants wishing to continue using contraception after the study will be referred for ongoing contraceptive care within the routine health services.

CONFIDENTIAL

**Table 5: Final Visit Procedures**

| Component | | Procedures |
|---|---|---|
| **Administrative and Regulatory** | | • Review/update locator information<br>• Provide reimbursement |
| **Behavioural/Counselling** | | • Assess sexual risk behaviour including sexual practices, condom use, etc.<br>• Provide counselling:<br>− method related side effects and continuation<br>− HIV/STI risk reduction including condom use |
| **Clinical** | | • Assess for adverse events (section 9)<br>• Collect contraceptive use information<br>• Provide contraceptive injections, as indicated/desired<br>• Conduct pelvic exam<br>• Verify the presence of IUD or implant<br>• Conduct syndromic assessment/treatment for STIs/RTIs |
| **Laboratory** | Urine | • Test urine for pregnancy[1] |
| | Pelvic | • Collect endocervical specimens for GC/CT testing<br>• Collect endocervical specimen for archiving<br>• Collect cervicovaginal specimen for semen exposure testing |
| | Blood | • Test blood for HIV (rapid test)<br>• Collect specimen for archiving |

[1]If a woman has had a clinician verified miscarriage or pregnancy termination within 12 days, a urine pregnancy test is not required

### 5.7.4 Possible HIV seroconversion visit

If HIV seroconversion is suspected, in addition to final visit procedures (though women who become HIV positive will remain in the study to the end of the follow-up period, sites will proceed with a physical exam, confirmatory testing (Western Blot and/or HIV EIA, with HIV RNA PCR) and CD4 testing (Appendix 7). Sites will schedule women with possible HIV seroconversion for a follow-up visit one to two weeks later for a review of HIV test results and also for the next regular visit.

### 5.8 Unscheduled Visits

Sites will ask women to return to the study clinic with any problems or concerns, including concerns about contraceptive complications, adverse events, suspicion of pregnancy, or general concerns. Study staff will document the reason for the unscheduled visit.

CONFIDENTIAL

**5.9 HIV Prevention Package**
At each visit, study staff will counsel women on HIV risk reduction including use of dual methods for prevention of pregnancy and STI, provide HIV testing and screening, syndromic treatment for STIs and offer them male and/or female condoms. We will also offer partner testing and counselling to any study participant that desires it including women who are in discordant partnerships. HIV serodiscordant couples will be counseled about ART for HIV risk reduction and referred for ART initiation at local service partners. If, during the course of the study, other new effective prevention strategies are incorporated into national HIV prevention policies (e.g., oral pre-exposure prophylaxis), study participants will be counselled about these interventions, and referred to local centres with appropriate expertise, in accordance with WHO/UNAIDS guidelines and local practice and stakeholder consultation. Thus, both post- and pre-exposure prophylaxis will be offered, either on site or by referral, per local standard of care /national policy. The study team is committed to ensuring that participants are aware of and have the option to access optimal HIV prevention tools, in accordance with local practice and standards. Use of HIV prevention strategies will be recorded on case report forms.

**5.10 Primary HIV Endpoint Determination**
All study sites will test for HIV per the algorithm shown in Appendix 7 for purposes of primary endpoint determination. Trained personnel will do the testing in accordance with the manufacturer's instructions. Two rapid tests will be done in parallel; if either (or both) test is positive ("possible seroconversion"), confirmatory testing will be completed. The specific rapid tests used will be determined by availability at each participating site; brands of tests used must be approved for use by national HIV testing policies. Each site will participate in proficiency testing throughout the study period. HIV testing will be accompanied by counselling in accordance with national HIV counselling and testing guidelines.

In the event of possible seroconversion, confirmatory testing will be initiated, specifically HIV Western blot and/or HIV EIA, with HIV RNA PCR. Negative or indeterminate confirmatory testing results will trigger core clinical safety/laboratory consultation to ensure timeliness and accuracy of testing and interpretation. Additional testing, including repeat Western blot and HIV RNA as well as HIV DNA PCR, may be initiated on archived samples collected at the time of possible seroconversion or on additional samples collected at a visit ideally 1-2 weeks after the possible seroconversion visit. An endpoints committee will review and confirm all possible seroconversion results and related laboratory testing. In the event of possible or confirmed HIV seroconversion, follow-up and use of the randomised contraceptive method should not be stopped/discontinued unless requested by the study participant

**5.11 Specimen Collection and Processing**
All specimen transport, processing, storage, testing, and results reporting will be conducted in accordance with standards of good clinical and laboratory practice. Each study site will establish standard operating procedures, including specimen chain of custody and QC/QA procedures, for all protocol-specified laboratory tests before study initiation.

**5.12 Specimen Handling and Biohazard Containment**
As the transmission of HIV and other blood-borne pathogens can occur through contact with contaminated needles, blood, blood products, and other biologic specimens, all personnel will use appropriate precautions in sample collection, processing, transport, shipping and handling, as recommended by the United States Centers for Disease Control and Prevention and relevant national and international guidance. All relevant materials, including diagnostic specimens and infectious substances, will be transported according to instructions detailed in the International Air Transport Association (IATA) Dangerous Goods Regulations. Potentially biohazardous waste will be contained according to institutional, national, and other applicable regulations.



CONFIDENTIAL

### 5.13 Procedures for HIV Seroconverters and Ancillary Studies of Early HIV Disease

Sites will continue to follow confirmed HIV seroconverters on the study schedule, with all procedures except on-going HIV serologic testing and related pre- and post-test counselling. Participants who seroconvert can continue their study contraceptive methods unless contraindicated[65]. Plasma HIV RNA and CD4 quantification will be obtained in the event of possible seroconversion at all subsequent quarterly visits and at the final study visit. Potential additional sample collection for studies of early HIV disease will be included, dependent on site capacity, participant consent, and availability of funding and are outlined in Appendix 8. Sites will fully counsel participants who seroconvert and refer them to local HIV care providers for on-going care.

### 5.14 Procedures for ancillary studies of biologic correlates of HIV acquisition

Potential additional sample collection for studies of biological mechanisms associated with contraceptive initiation and HIV seroconversion and correlates of HIV acquisition will be included, dependent on site capacity, participant consent and availability of funding as outlined in Appendix 9. Collection of these samples for ancillary studies will not involve any additional study visits.

### 5.15 Retention of study subjects

Once a participant is enrolled in the study, the study team will make every reasonable effort to retain her in follow-up, to minimise bias associated with loss to follow-up. The study team will track retention rates and address any issues related to retention. All study sites are responsible for developing and implementing site specific SOPs to achieve this. Components of such procedures may include:

- Thorough explanation of the study visit schedule and procedures during informed consent, and re-emphasis at each study visit
- Encouragement of participants to discuss potential study participation with their husbands/partners and other influential family members before agreeing to enrol in the study.
- Collection of detailed locator information at screening, and review and updating of this information at each study visit
- Use of a participant tracker database to facilitate scheduling and tracking
- Use of appropriate and timely visit reminder mechanisms (including phone calls and text messages, if participants agree)
- Immediate and multifaceted follow-up on missed visits, including home or other off-site visits if agreed upon
- Mobilization of trained outreach workers to complete in-person contact with participants at their homes and/or other locations
- Having a group of trained outreach workers at each site meet on a regular basis with the Site Investigator or designee to review retention efforts.
- If a participant fails to appear for a scheduled visit, local study staff will contact her as soon as possible. All attempts to contact a participant will be documented in the participant's study file. A participant will not be considered lost to follow-up until her study follow-up period (18 months or until closure of the study or study site) has finished, regardless of the point at which the last visit occurred.

## 6. ASSIGNMENT OF INTERVENTIONS

### 6.1 Allocation

Random allocation will use a 1:1:1 allocation to the 3 arms of the trial, stratified by site. Random block sizes between 15 and 30 will be used.

CONFIDENTIAL

### 6.1.1 Sequence generation

Random sequences will be generated at the study data management centre using a pseudo random number generator (SAS, Inc.). Randomisation lists will be generated as a fixed order of allocation for each site, and will be generated during trial implementation.

### 6.1.2 Allocation concealment mechanism

Allocation to arm will be assigned at the time of randomisation using standardised procedures and a predetermined sequence that is concealed from all study staff prior to randomisation (e.g. opaque, numbered envelopes or on-line or telephonic randomisation system).

### 6.1.3 Implementation

The randomisation sequence will be used in sequential order. Date and time of randomisation will be witnessed and recorded on the study source documentation and study CRFs. The order of randomisation compared to the original list order will be used to verify strict order of randomisation.

### 6.2 Blinding

This is an open-label study – neither study staff nor participants will be blinded to treatment arms after the point of randomisation. Nonetheless, strict policies will be in place to preserve randomisation integrity. Randomisation documentation will be stored in a secure location. Laboratory testing, data recording, and other assessment of the primary endpoint will be blinded to treatment arm; secondary study endpoints laboratory testing, data recording, and other assessments will be blinded to treatment arm where possible. The DSMB will be provided with unblinded information in its closed study reports.

## 7. DATA MANAGEMENT

### 7.1 Data collection methods

Interviewers will collect participant data on study CRFs. As appropriate for the question, CRFs will be translated into local languages as necessary, with translations verified for accuracy by back translation. Clinical study staff will record clinical data, including all SAEs and method-related AEs resulting in method discontinuation, on study CRFs. Trained MedDRa coders will code AE data centrally. Clinic staff will record results from rapid HIV tests on CRFs. Confirmatory laboratory HIV testing will be recorded on study CRFs or via electronic file to the Statistical and Data Management Centre by the testing laboratory.

Every effort will be made to continue women on their randomly assigned contraceptive method. If method switching and/or discontinuation occurs, women will be continued on study, and timing of discontinuation and initiation of any new method documented in the study CRFs. Reasons for discontinuation will be recorded, including discontinuation because of AEs or pregnancy.

### 7.2 Data management responsibilities

Study CRF flow and site data management (e.g. CRF transmission, query resolution, etc.) will follow site data management SOPs.

Study CRF data will be transmitted electronically and managed throughout study conduct in a central database at the Statistical and Data Centre using the DataFax clinical trials system. Data quality checks for the protocol database are documented in a protocol specific management document developed by the Statistical and Data Centre that will be

CONFIDENTIAL

available on the study website. Data are recorded on paper CRFs, and entered into a central database via DataFax. QC reports from DataFax are used to manage and resolve data inconsistencies.

Local laboratory data will be entered on study CRFs. QCs reconciling results from local and central sources will be implemented at the Statistical and Data Centre. Final resolution of testing discrepancies in primary endpoints is the responsibility of the study endpoint committee.

## 8. STATISTICAL METHODS

### 8.1 Sample size

The primary objective of this randomized trial is to compare the risks of HIV acquisition among women assigned to DMPA, LNG implant, and the copper IUD. The rate of HIV acquisition in women assigned DMPA will be compared to that in women assigned the LNG implant and the copper IUD; importantly, neither the LNG implant nor the copper IUD is assumed to be a neutral control and both comparisons are of interest. In addition, given the limited data available related to HIV risk and contraceptive methods other than DMPA, the LNG implant versus copper IUD comparison is also of scientific and public health importance. In estimating the sample size required for this trial, both the potential for correctly concluding that increased HIV risk exists (i.e., statistical power) and for falsely concluding increased risk when risk does not truly exist (i.e., type I error) have been considered in depth. The strategy detailed below balances statistical power, type I error control, and study size in this context of a trial with no placebo arm and three comparisons of interest.

This study is designed to have 80% power to detect 50% pair-wise increases in risk of HIV acquisition among women randomised to three different contraceptive methods. Each of the three individual comparisons will be made using a two-sided type I error of 0.04 (more stringent than the traditional alpha of 0.05) to control the overall chance of one or more type I errors at the 0.10 level. Thus, there is only a 4% chance of falsely concluding that any two particular methods differ and less than a 4% chance of falsely concluding that any particular method (e.g., DMPA) increases risk versus either of the others. In addition, there is less than an 8% chance of falsely concluding that one or both hormonal methods differ (in either direction) from the IUD if all three are truly equivalent. As a further consequence of the design, if only one method increases risk by 50% then there is greater than a 90% chance of concluding it is harmful versus one or both other methods.

The estimated trial size is defined by the target of observing at least 250 incident HIV infections for each pairwise comparison, to achieve 80% power to detect a 50% increased risk with a two-sided type I error of 0.04. This calculation allows for up to 10% dilution of treatment effect due to loss of person time on allocated contraceptive method, e.g., method discontinuation. The total numbers of enrolled participants and HIV seroconversion events necessary to achieve these pairwise event totals will depend on the patterns of risk across the methods, the underlying incidence rate in the study population, and the outcome of monitoring procedures designed to drop one or more arms if there is compelling interim evidence of increased risk or lack of effect among the various methods. These considerations will be reviewed by the DSMB on an on-going basis. Taking into account this uncertainty, a total cohort size of approximately 7,800 has been selected, assuming an underlying incidence of 3.5 per 100 woman-years, 18 months of prescribed follow-up per woman and a maximum of 10% loss to follow-up or early discontinuation.

### 8.2 Statistical analyses

A detailed statistical analysis plan (SAP) that covers both the final analysis and planned interim analyses will be developed and approved prior to the first interim analysis. The following is a summary of the planned analyses. Any deviations made from this summary plan will be documented in the detailed SAP.



CONFIDENTIAL

#### 8.2.1 Primary analysis

The primary study objective is to compare the risks of HIV acquisition among women randomised to DMPA, LNG implant, and the copper IUD. The primary analysis will include computation of the hazard ratios of HIV seroconversion based on a proportional hazards (PH) regression model, stratified on site. Only contraceptive method group will be included in the primary stratified model. An appropriate interim spending function will be used to account for interim analyses and control the type I error for each comparison at the 0.04 level. Women will be analysed according to their randomised contraceptive method, regardless of method switching; only women who are found to have been HIV-infected at enrolment (defined by a positive HIV RNA PCR test on the archived enrolment plasma sample) or who fail to contribute a follow-up HIV test result will be excluded from the primary analysis. Women who do not HIV seroconvert during the study will have their time in analysis censored at the date of their last negative HIV test result.

#### 8.2.2 Analysis of secondary endpoints

**Pregnancy rates**

The study will compare the risks of pregnancy between method groups based on two-sided 95% confidence intervals for the hazard ratios of pregnancy obtained from a PH regression model, stratified by site. The study team will analyse pregnancy rates both according to randomised contraceptive method group (primary) and restricted to periods with exposure to each contraceptive method (secondary). If too few pregnancies are observed in a particular group to justify asymptotic methods, exact log-rank tests will be used to make comparisons at the two-sided alpha=0.05 level of significance.

**Contraceptive method continuation**

The study will summarise method continuation in frequency tables including reasons for discontinuation (e.g. refused assigned method, AE-related, participant request to stop or switch methods). The study will assess differences across randomisation groups using exact tests conducted at the two-sided 0.05 significance level. The study will compare rates of method discontinuation or switching (time to first event) between randomisation groups using two-sided, 95% confidence intervals for hazard ratios obtained from a stratified PH model. Cumulative probabilities of remaining on assigned method will be estimated based on Kaplan-Meier survival methods.

**Adverse events**

AEs that are either serious or result in method discontinuation will be summarised in frequency tables (including both the number of each type of AE and the number of distinct participants ever reporting each type of AE), by system organ class and preferred MedDRA term.

The study will compare rates of SAEs between contraceptive groups using two-sided 95% confidence intervals based on exact Poisson methods. Results will be reported overall, by relatedness, and by geographical region. The analysis will be conducted both according to randomised contraceptive method group and restricted to periods with exposure to each contraceptive method.

The study will similarly compare rates of AEs resulting in method discontinuation between groups using two-sided 95% confidence intervals. This analysis will exclude women who never adopted their assigned method, and will be based on time to first method discontinuation.

#### 8.2.3 Analysis of tertiary endpoints

**Baseline age and HSV-2 status as modifier of the hormonal contraception and HIV acquisition relationship**

CONFIDENTIAL

The study team will use survival analysis methods that parallel the approach used in the analysis of the primary outcome to assess these tertiary objectives. For each objective, a PH regression model will be fit with contraception arm as covariates, incorporating interactions between baseline age category or HSV-2 status and method group. All interactions will be assessed at the two-sided 0.05 level of significance.

**Early HIV disease progression**

Log-transformed viral load (VL) and CD4 T-cell count data will be compared between contraceptive groups among women who seroconvert using mixed effects models to account for repeated measures on subjects. Analyses will be performed according to women's original allocated method and according to contraceptive method used at the visit prior to seroconversion. Summaries will include absolute VL and CD4 measures from the seroconversion visit onward, as well as change in VL and CD4 from the serocoversion visit.

**8.2.4 Methods for any additional analyses**

**Analysis of subgroups**

HIV seroincidence and pregnancy analyses will be conducted according to region, site, age, body mass index (BMI), coital frequency, marital status and a limited number of other subgroups specified in the SAP. All subgroups analyses will be performed using descriptive two-sided 95% confidence intervals.

**Analysis of cofactors**

The study will explore the potential effects of cofactors on the rate of HIV infection using multivariate PH regression models. Details of these exploratory analyses, including a list of baseline and time-varying covariates which will be considered for analysis, will be provided in the SAP.

**Per protocol and as treated analyses**

Because there is potential for method refusal after allocation, contraceptive switching and discontinuation to affect the results of the trial, both a per-protocol and an as-treated analysis will be performed and presented with the primary results. For the per-protocol analysis women will be censored at the point they stop using (or never initiate) their assigned contraceptive method (including discontinuation due to pregnancy). For the as-treated analysis, women will be analysed according to the method they initiate, and will account for any subsequent method switching to study groups using time-varying treatment effects (women who cease contraception or switch to a non-study method will be censored from the as-treated analysis when either occurs). The potential for selection bias and confounding to influence per protocol and as-treated analyses will be explored using appropriate estimation techniques (e.g. inverse probability weighting). Details of these analyses, including potential confounders to be measured and definitions of adherence criteria, will be provided in the SAP.

**8.2.4 Methods for handling missing data**

Primary HIV analyses will censor participants who are lost to follow-up at the date of their last known HIV status. Other missing data will be ignored (i.e. considered missing completely at random), so long as the prevalence of missing outcomes is less than 3%. If the rate of missing data is higher than 3% then multiple imputation methods will be used in estimation and statistical inference of secondary objectives.



CONFIDENTIAL

### 8.2.5 Interim Analysis Plan Summary

An independent Data and Safety Monitoring Board (DSMB) will convene approximately every six months to assess safety and operational metrics (including accrual, method continuation, retention, and HIV incidence rates). Formal interim analyses designed to assess the relative effects of contraceptive method use on risk of HIV will occur after approximately 25%, 50% and 75% of the expected event totals have been reached, as detailed in the DSMB Charter. Although the DSMB may recommend that the trial be modified or halted based on various sources and strengths of evidence, pre-specified guidelines founded on sound statistical principles provide formal justification for making such changes. To that end, it is anticipated that a method group may be discontinued from further study if it is associated with a significant increased risk of HIV (compared to either other method) when controlling the type I error rate at the two-sided 0.04 level using appropriate stopping boundaries. Follow-up of the remaining methods may continue unless there is compelling evidence that none are associated with a 50% increased risk. Without early stopping, it is anticipated that an estimated hazard ratio of approximately 1.3 or larger would be considered statistically significant at the final analysis.

## 9. MONITORING

### 9.1 Data monitoring: Data and Safety Monitoring Board

This study will be subject to oversight by a DSMB that will complete periodic review of study conduct and interim analyses of study safety and HIV acquisition data. The DSMB will be an independent group of experts that will advise the funders and the study team. The DSMB will consider study-specific data as well as relevant background knowledge about the disease, study interventions, and/or population under study. The DSMB will maintain the confidentiality of its internal discussions and activities as well as the contents of reports provided to it. The DSMB will be established prior to trial initiation and will operate under a charter that will be written in coordination with the DSMB. Additional information about the DSMB composition and review procedures will be contained in that separate DSMB charter. The DSMB charter will define approaches for consensus development and other aspects of the DSMB operations.

The primary responsibilities of the DMSB are to 1) periodically review and evaluate the accumulated study data for participant safety, study conduct and progress, and HIV acquisition risk, and 2) make recommendations to the study investigators and the sponsor concerning the continuation, modification, or termination of the trial. At each review, the DSMB may recommend that the study proceed as designed, proceed with design modifications, or be discontinued. Summaries presented to the DSMB will include participant accrual rates, comparability of the study treatment groups at enrolment, method continuation/discontinuation and switching, loss-to-follow-up, adverse event information, and HIV incidence. Reports from open sessions of DSMB meetings will be provided to study sites for their information and for submission to their IRBs/ECs.

Following study initiation, the DSMB will review data on safety, study conduct, and scientific validity and integrity of the trial approximately every 6 months. Periodic interim reviews of HIV incidence, at the point at which these are planned, will coincide with scheduled meetings, or, at the discretion of the DSMB, may occur separately at unscheduled times.

#### 9.1.1 Monitoring Quality of Study Conduct Operational Characteristics and Implementation

Before the study starts, the study team will define goals for key operational metrics, including study recruitment (e.g., enrolment rate, screen to enrol ratio), retention (percentage of expected visits completed, participant withdrawal and loss to follow-up), and method discontinuation (refusal at the time of randomization to accept as-randomized method, discontinuation of randomized method, and percentage of person-time not on randomized method). On a regular basis, the Statistical and Data Centre will report operational metrics to the operations team and the operations team will

CONFIDENTIAL

communicate with study sites concerning operational performance according to these metrics. The approach will emphasize transparency regarding these critical measures of study success.

Goal measures for operational metrics – e.g., enrolment rate on pace to achieve the target sample size within approximately 18 months, low rate of refusal of randomized method at the time of randomization, rates of method discontinuation that are sufficiently low as to not jeopardize trial integrity – will be defined in the trial's DSMB Charter and will be discussed between the study team, funders, and DSMB ahead of trial initiation. The DSMB Charter will acknowledge that both individual metrics (e.g., high retention) and combinations of metrics (e.g., high retention, with high method continuation) are important to the success of the trial. The study team, funders, and DSMB will monitor study operations, and may agree to terminate the trial entirely or modify the study (e.g., discontinue individual arms of the trial) for poor accrual/recruitment, low method continuation, poor retention, and/or low HIV incidence. Regular discussions between the study team, funders, and DSMB, at least coincident with scheduled DSMB reviews and additionally on an ad hoc basis as required, will address whether changes to study execution or design are required. The Statistical and Data Centre will provide regular reports that outline the potential impact on the study's ability to detect a difference between the treatment arms if there are deviations from the statistical design in terms of accrual/recruitment, method continuation, retention, and/or HIV acquisition. The DSMB will make recommendations regarding adjustment of sample size or other aspects of the study design, if required.

### 9.1.2 Monitoring of Safety and HIV Acquisition Endpoints

Before the study starts, the study team, in conjunction with the DSMB, will develop a DSMB Charter that will provide details on the interim monitoring strategy for HIV and safety endpoints.

Using the plan as guidance, the DSMB may recommend early termination of the study or modification (e.g., discontinuation of an individual arm) if there is clear evidence of harm or operational futility with respect to one or more of the study arms. The study team, funders, and DSMB will discuss the potential for adaptations in the continuation of the trial in response to interim reviews (e.g., discontinuation of one arm in response to elevated HIV risk, with continuation and potential extension of follow-up in other arms). The type I error for each method group comparison will be controlled at the two-sided $\alpha=0.04$ significance level using group sequential stopping boundaries. The information level for each comparison will be computed according to the interim observed and expected total number of primary endpoints at each interim analysis. The DSMB will also assess non-HIV safety information, according to endpoints defined in the secondary aims of the study protocol. The DSMB may request additional analyses of the safety and/or HIV acquisition data.

## 9.2 Safety Monitoring by Protocol Team

The study site investigators are responsible for continuous safety monitoring of all study participants, and for alerting the safety monitor and protocol management team if unexpected concerns arise. The safety monitor and protocol management team will review participant safety data regularly throughout the course of the study. To support this monitoring, the study statistician will prepare study progress reports and tabulations of reportable adverse events (AEs, defined below) experienced by participants for review. Protocol team members will routinely review these reports and discuss potential participant safety issues throughout the period of study implementation.

### 9.2.1 Adverse Events (AEs)

Participants will be provided instructions for contacting the study site to report any untoward medical occurrences they may experience, except for possible life-threatening events, for which they will be instructed to seek immediate emergency care. Participants will be able to seek evaluation at the study site, where feasible and medically appropriate.


CONFIDENTIAL

With permission of the participant, and whenever possible, records from all non-study medical providers related to untoward medical occurrences will be obtained for review. All serious adverse events (SAEs) and AEs resulting in method discontinuation will be record on study CRFs. Trained MedDRA coders will code AE data centrally. Adverse events will be followed until resolution or until the participant's final study visit; participants who have on-going adverse events at their last study visit will be referred for additional care.

### 9.2.2 Serious Adverse Events

SAEs will be defined as any AE that:

- Results in death
- Is immediately life-threatening
- Requires inpatient hospitalization or prolongation of existing hospitalization
- Results in persistent or significant disability/incapacity
- Is a congenital anomaly/birth defect
- Is an important medical event that may not result in death, be immediately life-threatening, or require hospitalization but may jeopardise the participant or require intervention to prevent one of the outcomes listed in the definition above

### 9.2.3 Adverse Event Relationship to Study Product

The on-site investigator/designee will assess whether an AE is related to the study agent using the available information about the study products and his/her clinical judgment.

The site investigator/designee has authority to stop the contraceptive method for any participant if significantly concerned about clinical or laboratory AEs. The decision to discontinue an assigned contraceptive method as a result of an AE will be immediately communicated to the study operations team. Relationship to study methods will also be reviewed centrally.

### 9.2.4 Adverse Event Reporting Requirements

Sites will monitor AEs starting at randomisation and continuing through the final study visit. Study site staff will document in source documents all AEs reported by or observed in enrolled study participants regardless of severity and presumed relationship to study product. Only AEs that are either a) serious or b) resulting in method discontinuation will be recorded on CRFs. Relevant AEs will be reported to site regulatory authorities according to local guidelines.

Study sites will report all SAEs, including those that may appear to be unrelated to the study interventions (contraceptive method), to the medical monitor at the University of Washington, within 48 hours of becoming aware of the problem. Additional information about these SAEs, including copies of outpatient records, hospitalization summaries, pathology reports, operative reports, and laboratory reports, may be requested. SAEs will be reported to overseeing Institutional Review Boards (IRBs)/Ethics Committees (ECs) according to their policies.

### 9.3 Social Harms Reporting

Although study sites will make every effort to protect participant privacy and confidentiality, it is possible that participants' involvement in the study could become known to others and that social harms may result. For example, participants could be treated unfairly, or could have problems being accepted by their families, partners and/or communities.



CONFIDENTIAL

Sites will assess for study-related social harm at each scheduled follow-up visit; reports can also be filed at interim visits if study-related social harms are discovered between visits. Information regarding social harms related to study participation will be recorded into the study database. Participants who report social harms will be referred to speak with a study counsellor and, if appropriate, a study clinician and the site investigator/designee. Sites may refer participants to appropriate additional resources for safety as needed.

If a site investigator/designee judges a social harm related to study participation to be serious and unexpected, the study safety monitor and responsible site ECs/IRBs (according to their individual requirements) will be notified. While maintaining participant confidentiality, study sites may engage their community advisory boards (CABs) in exploring the social context surrounding instances of social harm.

**9.4 Regulatory Requirements**

The Investigator will maintain and store in a secure manner complete, accurate, and current study records throughout the study. The Investigator will retain all study records as authorized by the operations team in compliance with local regulations. Study records include administrative documentation —including protocol registration documents and all reports and correspondence relating to the study —as well as documentation related to each participant screened for and/or enrolled in the study —including informed consent forms, locator forms, case report forms, notations of all contacts with the participant, and all other source documents.

The trial will be conducted in compliance with the protocol, GCP, and the applicable regulatory requirement(s) of the US and study site countries. In addition, FHI 360 will be responsible for maintaining central documentation to demonstrate GCP compliance.

**9.4.1 Quality control and quality assurance**

The study team will conduct regular quality control visits to ensure protocol compliance and discuss operational issues or problems. This team will likely include one or more operations team member, a clinical trainer, and a clinical research manager. Areas to be evaluated and discussed may include study site staffing concerns, ways to target the appropriate study population, minimizing of method discontinuation, improving study retention, and issues with clinical or contract laboratories, financial and administrative concerns, etc.

**9.4.2. Source documents and access to source data/documents**

Sites will maintain appropriate source documents, as detailed in the study MOP. Source documents will be access controlled and limited to appropriate study staff and monitors/auditors.

**9.4.3. Protocol deviations**

Site investigators and staff must follow the protocol in entirety, without deviation. Departures from the protocol must be authorized by the site investigator or designee, and are allowable only if necessary to eliminate an immediate hazard to a study participant. Protocol deviations will be documented on case report forms and reported to the governing IRBs as required.

**9.5. Clinical Site monitoring**

Qualified independent clinical monitors will conduct periodic study monitoring in accordance with FHI 360 policies. Before the study begins, FHI 360, in conjunction with the independent monitors, will develop a detailed clinical monitoring plan. Briefly, the study clinical monitors will:

- Review informed consent forms, procedures, and documentation

CONFIDENTIAL

- Assess compliance with the study protocol, Good Clinical Practices (GCP) guidelines, and applicable regulatory requirements (US and non-US)
- Verify all SAEs in source documents have been reported on CRFs and in accordance with regulatory guidelines
- Perform source document verification to ensure the accuracy and completeness of study data
- Verify proper collection and storage of biological specimens
- Verify proper storage, dispensing, and accountability of study products
- Assess implementation and documentation of internal site quality management procedures
- Verify that current license/certification is available on site for study staff listed on the current Statement of Investigator Form, and Delegation of Responsibilities Log/Form.

The Site Investigator or designee will allow study monitors to inspect study facilities and documentation (e.g., informed consent forms, clinic and laboratory records, other source documents, CRFs), as well as observe the performance of study procedures. Site investigators will also allow inspection of all study-related documentation by authorised representatives of the Consortium; OHRP and local and US regulatory authorities. Sites will maintain a site visit log to document all visits.

### 9.6 Auditing

The investigators and sites may be subject to a field audit by the sponsor (FHI 360, WHO and/or designees) or regulatory agencies. Such an audit could occur while the study is in progress, several years after the study is completed, or when the data are under review by a regulatory agency. All participant records and other study documentation must be filed and accessible on short notice (three to five days) during the study and subsequent retention period. Documents should be adequate to reconstruct the course of study events. Study documents need to be retained for a minimum of 5 years until authorized in writing by the ECHO operations team.

## 10. GOOD PARTICIPATORY PRACTICE

The Good Participatory Practice (GPP) guidelines were created in 2007 by UNAIDS and AVAC, an HIV prevention research advocacy group, to set global standards in stakeholder engagement for biomedical HIV prevention trials. Good participatory practice during the entire life-cycle of an HIV prevention trial can help ensure that research is relevant and meaningful and may also enhance the quality and outcomes of clinical research.

The ECHO trial will use the most recent edition of the GPP guidelines (2011) to guide the study's engagement with site communities and local, national, regional and global stakeholders. According to the GPP guidelines, "community stakeholders" include both individuals and groups that are ultimately representing the interests of people who would be recruited to or participate in a trial, and others locally affected by the trial. These may include trial participants, people living in the area where the research is being conducted, people in the area who are infected and affected by family planning needs, reproductive health issues and HIV, local non-governmental organizations, community groups and community-based organizations [66].

Good participatory practice in HIV prevention research goes beyond having a Community Advisory Board (CAB). It involves utilizing a range of stakeholder advisory mechanisms to promote transparent, meaningful, collaborative, and mutually beneficial relationships with all levels of stakeholders (See Figure 1). The ECHO trial will strive to embrace the GPP guiding principles throughout the implementation of the study. These include: respect, mutual understanding, integrity, transparency, accountability and community stakeholder autonomy.



CONFIDENTIAL

**Figure 1: Examples of Stakeholder Advisory Mechanisms from GPP Guidelines, 2011**

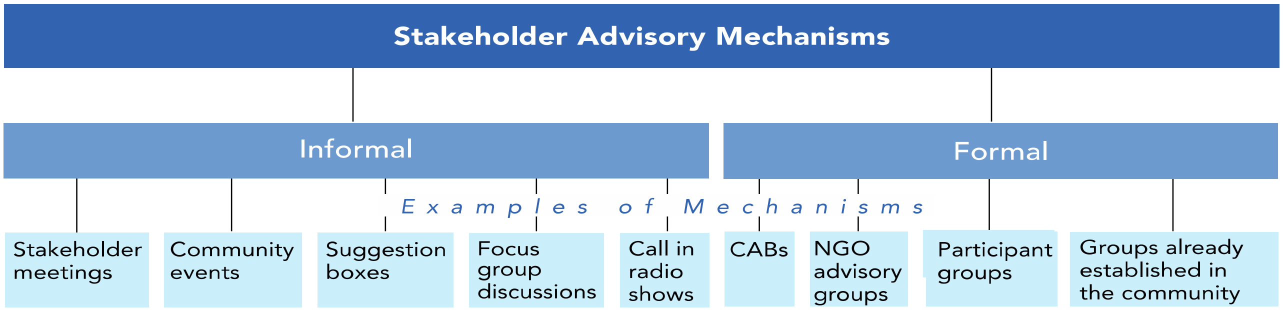


Stakeholder advisory mechanisms take many forms. This figure shows that they can be informal or formal and provides several examples of each type of stakeholder advisory mechanism. All of these mechanisms, as well as others, may be used to facilitate important dialogue between research teams and other stakeholders.

The ECHO team is committed to conducting meaningful community consultation, from protocol development and implementation to results dissemination and policy development where appropriate. To facilitate effective implementation of the GPP Guidelines, all members of the study team will be trained on the GPP Guidelines. Resources will be developed and technical support for GPP activities will be provided to encourage and facilitate meaningful stakeholder engagement at all levels of study implementation. Details of tools and time lines for implementing the GPP Guidelines will be outlined in the study procedures manual [66].

## 11. ETHICS AND GENDER

### 11.1 Institutional Review Boards/ Ethics Committees

Ethical approval will initially be obtained from the Protection of Human Subjects Committee (PHSC) of FHI 360, the Research Project Review Panel (RP2) of the Human Reproduction Program of the Department of Reproductive Health and Research at the World Health Organization, and from the Ethics Committee (EC) of the World Health Organization. Each participating site will also seek approval from the appropriate local Institutional Review Boards. Copies of the submissions and approvals should be retained in the site's regulatory file and the main trial master file.

Prior to implementation of this protocol, and any subsequent full version amendments, each site must have the protocol and the protocol consent forms approved, as appropriate, by their local IRB/EC and any other applicable regulatory entity.

### 11.2 Protocol Registration

The study team will register this trial on clinicaltrials.gov, a registry database of clinical studies of human participants conducted around the world, and updated at regular, specified intervals. In addition, the trial will be registered with the World Health Organization as part of their clinical trials database.

### 11.3 Risk Benefit Statement

#### 11.3.1 Risks

Site investigators will make efforts to minimise risks to participants. It is not expected that this trial will expose human subjects to unreasonable risk. Pelvic examination and procedures may cause mild discomfort and/or vaginal bleeding or

CONFIDENTIAL

spotting. Phlebotomy may lead to discomfort, feelings of dizziness or faintness, and/or bruising, swelling and/or infection. Women who learn they are HIV-positive, or have a reproductive tract infection, in the course of the trial may experience worry, sadness or depression. Trained counsellors will be available to help participants deal with these feelings. Participation in clinical research includes the risks of loss of confidentiality and discomfort with the personal nature of questions when discussing sexual behaviours.

Participants at sites requiring partner notification in response to diagnosed STI or HIV infection could have problems in their relationships with their sexual partners. Participants also could have problems in their partner relationships associated with the use of a contraceptive method.

Site staff will make every effort to protect participant privacy while in the study. Although study sites make every effort to protect participant privacy and confidentiality, it is possible that participants' involvement in the study could become known to others, and that social harms may result (i.e., because participants could become known as at "high risk" for HIV infection). For example, participants could be treated unfairly or discriminated against (see Section 9.3 for details).

The risks specific to the contraceptive methods used in this study are low, but not non-existent. IUD users are at risk of uterine perforation (1/1000), pelvic inflammatory disease in the first 20 days after insertion, or expulsion of the device (5% in the first year of use); all of these complications are rare. In addition, IUD insertion is uncomfortable for most women, although generally symptoms resolve within ten to fifteen minutes; symptoms are usually completely resolved within one day after insertion. Users of DMPA may experience irregular bleeding, amenorrhea, a small amount of weight gain, long return to fertility, or pain at the injection site. Users of contraceptive implants may experience irregular bleeding, amenorrhea, or pain at the insertion site. Contraceptive failure is possible with any of the methods, although when used correctly and consistently all of the study methods are over 99% effective in preventing pregnancy. The contraceptive-related risks anticipated to be experienced by participants in the study are no different from those that would be anticipated among contraceptive users who were not participating in the study.

Although the potential for increased risk of HIV acquisition among users of progestin-only methods is hypothesized, whether such a risk indeed exists, and the degree of risk, are both unknown. Because of the predominance of progestin-only contraceptives in much of sub-Saharan Africa, if they did not participate in the study most participants would be using an injectable contraceptive, or no contraceptive at all.

### 11.3.2 Benefits

Participants will receive HIV/STI risk reduction counselling, HIV and STI testing, examination, and routine laboratory testing. Participants will be provided STI treatment in accordance with local standards of care free of charge. For other medical conditions identified as part of the study screening and/or follow-up procedures, participants will be referred to other sources of care available in their community. Some volunteers may have the opportunity to access expedient treatment and decreased morbidity due to early diagnosis and treatment of abnormalities identified during tests, examinations and referrals. Participants who are found to be HIV-positive in the course of the trial may benefit from earlier referral to care and, where indicated, treatment, than they may have had they not been participating in the trial.

Participants and others may benefit in the future from information learned from this study. Specifically, information learned in this study may lead to improved understanding about the safety of progestin-only contraceptives for women at high risk of HIV. Participants may appreciate the opportunity to contribute to the field of HIV and contraceptive research.

CONFIDENTIAL

### 11.4 Informed Consent Process

Written informed consent from all prospective study participants will be obtained prior to both screening and enrolment. Written consent (signed and/or witnessed) will be obtained by the Site Investigator or his or her designees. In addition, during recruitment activities that occur prior to screening and enrolment, verbal consent will be obtained from potential study participants before they are asked personal questions to determine their eligibility to enrol in the study. Written IC also will be obtained for long-term specimen storage and possible future testing as well as for off-site visits as needed.

Neither consent for long-term specimen storage nor off-site study visits are required for study participation. Any potential future use of stored specimens would be done without identifying information and via submission of a protocol to the appropriate ethics and regulatory authorities. Women will also be asked if they are willing to be contacted for any potential future studies. The Site Investigator shall seek consent under circumstances that provide the prospective participant with sufficient opportunity to learn about the study and consider whether or not to participate in it. IC must be obtained without coercion, undue influence or misrepresentation of the potential benefits and risks that might be associated with study participation. Literacy will be assessed prior to administration of written informed consent; details of assessment will be outlined in the MOP. In obtaining and documenting informed consent, the Site Investigator and their designees will comply with applicable local and US regulatory requirements and will adhere to GCP and to the ethical principles that have their origin in the Declaration of Helsinki. Study staff must document the informed consent process in the clinical notes, as per details outlined in the study-specific procedures manual. Participants will be provided with copies of the informed consent forms if they are willing to receive them.

In addition to informed consent forms, the study operations team will work with study staff and community representatives to develop appropriate materials about the study and a standardised approach to the informed consent process to be implemented at all study sites, which will be detailed in the study-specific procedures manual. In short, the entire process will be conducted in a private setting whereby study staff can review the entire consent with the potential participant and ensure sufficient understanding of the study and details covered in the consent prior to signing. All information, including the IC, will be translated into local language(s) as appropriate, in order for materials to be available in a language that is understandable to the participant. Back-translations and verifications will be done and copies maintained in the site regulatory files and the study master file.

The informed consent process will cover all elements of informed consent required by research regulations. In addition, the process specifically will address the following topics of importance to this study:

- Randomisation and the importance of participants in all study groups to the success of the study
- The importance of adherence to the study visit and procedures schedule
- The potential medical risks of study participation (and what to do if such risks are experienced)
- The potential social harms associated with study participation (and what to do if such harms are experienced)
- The real yet limited benefits of study participation
- The distinction between research and clinical care
- The right to withdraw from the study at any time

CONFIDENTIAL

### 11.4.1 Informed Consent for Minors

We will follow national guidelines and local IRB guidance for obtaining informed consent for minors and, where required, study staff will seek parental/guardian consent as well as assent from the minor participant. Where a previously pregnant 16-17 year old is considered to have reached the age of majority (i.e. is not considered a minor), we will seek informed consent directly from the young (16-17 year old) woman. Written informed consent and assent (where necessary) will be obtained prior to both screening and enrolment in accordance with national and local guidelines and regulations.

### 11.5 Participant Confidentiality

Site staff will conduct all study procedures in private, and will protect participant privacy and confidentiality to the extent possible. Study-related information will be stored securely at the sites. Sites will maintain any records that contain names or other personal identifiers, such as locator forms and informed consent forms, securely with limited access. Sites will also secure forms, lists, logbooks, appointment books, and any other listings that link participant numbers to identifying information in a secure area with limited access. Laboratory specimens, data collection, and administrative forms will be identified only by coded number and will also be kept secure, with access limited to study staff. Sites will protect any on-site databases with password access systems.

Participants' study information will not be released without their written permission, except as necessary for review, monitoring, and/or auditing by the following:

- Representatives of the US Federal Government, including the US OHRP, representatives of the US and host government and other local and US regulatory authorities
- Study management staff
- Site staff
- Site and central IRBs/ECs
- Sponsors
- Contractors working on behalf of the trial management

### 11.6 Compensation

Pending IRB/EC approval, participants will be compensated for time and effort for participation in this study. Reimbursement will cover transportation costs and time away from work, and may vary by site depending on local ethical review requirements. Site specific reimbursement amounts will be specified in the study informed consent forms and reviewed with participants upon study enrolment.

To encourage continued participation in the study, participants who remain in the study and follow study procedures may be eligible for additional reimbursement, and/or gifts depending on local IRB/EC requirements. No compensation will be contingent upon continuation of the allocated contraceptive method.

### 11.7 Deception

No deception will be employed in this study. All participants will be aware of the treatment arm to which they have been allocated and will be fully informed of the purpose of the study.

CONFIDENTIAL

**11.8 Protocol Amendments**

If changes are needed to the protocol, the protocol implementation team (in accordance with the protocol design team) will handle all changes centrally. Once the changes are finalised, the protocol will need to be submitted and approved by the appropriate IRBs and regulatory agencies prior to implementation. Version control will be maintained centrally and sites will be trained on any changes.

**11.9 Declaration of interests**

There are no conflicts of interest to declare. However, prior to study start all investigators will be asked to complete a conflict of interest and financial declarations form to be filed with the master trial files.

**11.10 Ancillary and post-trial care**

At scheduled and unscheduled visits, the participants will receive free routine clinical assessment, treatment for STIs or other conditions that can be handled within the clinic, and referrals needed for medical issues beyond the scope of the clinic. At the final visit, the women will be re-counselled about their contraceptive choices. Any women who wishes can have their study assigned method removed (IUD or implant) and can have any new study method provided at no cost. All study participants wishing to continue using contraception after the study will be referred for ongoing contraceptive care within the routine health services.

Participants who acquire HIV during the study will be managed at the research site or referred for management according to the local standard of care. Written SOPs for referral for HIV care and treatment will be in place at each study site, and these documents will identify appropriate referral options for persons with HIV infection, including medical care, antiretroviral therapy (ART), and psychological and social services. Some of the research sites will be part of health care institutions that provide HIV care and support, and can refer women to those services; other sites will have established referral agreements with programs providing HIV care services. Laboratory results obtained as part of study procedures (e.g., CD4 counts) may be helpful for clinical management and copies will be provided to the participant and her medical provider. At the conclusion of study participation, final care plans will be established for all HIV seroconverters to ensure on-going access and linkage to HIV care.

At the final visit, sites will ask participants whether they wish to be informed of the results of the trial. Those who wish to will provide contact information so that they can be notified of the results. Site will also provide women with contact information for the study clinic should they have questions after their participation is complete.

**11.11 Gender Considerations**

**11.11.1 How the project responds to a public health need affecting men or women**

Although maternal mortality has decreased in recent years, it remains unacceptably high, particularly in sub-Saharan Africa. Contraception is a significant contributor to preventing maternal death by reducing unintended pregnancy and allowing women to space or limit births. Improved access to contraception additionally helps women avoid unintended pregnancy and unsafe abortion and allows them to provide better for their families.

In sub-Saharan Africa, the majority of people living with HIV are women, and increasingly maternal deaths in regions with high HIV prevalence have been attributed to HIV. This project recognises that women require protection both from pregnancy and from HIV and seeks to determine if commonly-used contraceptives may put women at increased HIV risk. Both prevention of HIV and unintended pregnancy are critical public health needs affecting women.

CONFIDENTIAL

### 11.11.2. How the research contributes to reducing inequities between women and men in health and health care

The discrepancy between maternal mortality and morbidity rates for women living in low-income compared with high-income countries is persistent and problematic. The lack of access to a broad range of family planning services reflects women's lack of power and inequity in health care systems. Empowering women to make their own decisions about reproduction, including deciding when or if to have children, is central to promoting gender equity.

## 12. DISSEMINATION AND PUBLICATION POLICY

### 12.1 Applicability of Results

This trial will respond to an urgent need for clarity on a public health topic of particular importance in areas with high unmet need for contraception and high HIV incidence and prevalence. Due to the current uncertainty regarding the safety of progestin-only contraceptives for women at high risk of HIV infection, women, policy-makers, and clinicians are all left unsure about how to understand the evidence and women must be counselled about the concerns of a possible increased HIV risk, about the uncertainty over a causal relationship and about how to minimize their risk of acquiring HIV.. This trial seeks to resolve a high-priority reproductive health issue identified as critical by the WHO, policy-makers, researchers, and by women who are choosing among various effective contraceptive methods.

The results of this trial will inform the WHO's Medical Eligibility Criteria for Contraceptive Use by providing information about the safety of various effective contraceptive methods including progestin-only contraceptives for women at high risk of HIV. In turn, clinicians, policy-makers, and women will have more information at their disposal for contraceptive decision-making. This topic continually draws broad interest and exemplifies women's desires to prevent both HIV and unintended pregnancy. The knowledge gained in this research will be instrumental in shaping family planning services in areas of HIV incidence and will help ensure that those services provided are the safest available for women.

In addition to the knowledge gained from the primary outcome measure, HIV seroconversion, secondary outcomes will provide important information for contraceptive programming. For example, no study has directly compared the contraceptive effectiveness of these methods in a randomised design. This study will provide higher quality information to women about the comparative contraceptive effectiveness of these methods. Also, while it is known that women frequently discontinue or switch contraceptive methods, this study provides an opportunity to better quantify the frequency of discontinuation and switching, and to understand why women switch methods. Sub-studies of those women who seroconvert may provide valuable information on biological or social factors conferring vulnerability to HIV infection, which may impact future HIV prevention efforts.

### 12.2 Communication of trial results

When women enrol in the study we will inform them that they will be contacted at the end of the study so that the can be informed of the study results, if they so choose. In addition, we will share the trial results with the local community advisory board. Before public communication of the results, the team will also share study results with national ministries of health of participating countries. Results will be presented at relevant conferences on HIV, family planning and sexual and reproductive health, and published in peer-reviewed journals as open access articles (where feasible). The study team will include trial results in newsletters from WHO and FHI 360, which have broad readership in multiple languages and in the newsletters of participating African institutions where available. The study team will attempt to communicate the results to civil society groups with an interest in HIV, family planning and SRH by disseminating the results through networks of women living with HIV and networks of women's groups. Priority will also be given to communicating the results to national medical organizations in participating countries. Before results are publically released, media communication briefs will be developed for each country and for international journalists, and a select



CONFIDENTIAL

group of global and national journalists from participating countries will be confidentially briefed before the results are publicly announced. See section 10 above for more detail on good participatory practices that will ensure that those groups most in need of the information have access to it.

### 12.3 Access to data

While the trial is on-going, the study database will be kept confidential. After completion of the study, access to data will follow the requirements of the funding agencies and participating organizations.

### 12.4 Publication Policy

A Publications Committee will be formed to write a publication policy for the trial. Three levels of publications will be addressed in this policy:

- Primary publications: Those publications specifically reporting the results of the primary, secondary study, and tertiary objectives stated in the study protocol
- Secondary publications: Publications reporting on the study results using data from multiple study sites but not specifically addressing the stated objectives of the study
- Tertiary publications: Publications based on data from a unique study site and drafted by the study team at that site.

Authorship roles will be assigned primarily according to the contributions of individual members as well as the likelihood that the authors will be able to complete data analysis and manuscript preparation in a timely manner. All investigators will be encouraged to participate in discussions regarding authorship of primary and secondary publications. Abstracts and publications resulting from this study must be approved by the Publications Committee prior to submission to scientific meetings or journals.

### 12.5 Authorship eligibility

The ECHO management team will oversee authorship and publications. Eligibility for authorship will follow the International Committee of Medical Journal Editors recommendations for determining substantive contribution to a manuscript developed for publication.

## 13. MAIN PROBLEMS ANTICIPATED & PROPOSED SOLUTIONS

### 13.1 Main problems anticipated and proposed solutions

The protocol team has anticipated key potential threats to execution of this clinical trial, including slow enrolment, refusal of women to accept their allocated method, poor retention, and high contraceptive method discontinuation. Operational barriers will be addressed through careful selection and training of study sites, real-time monitoring of relevant metrics (e.g., enrolment rate, refusal to accept randomised method, loss to follow-up, method discontinuation), continuous process improvement activities and sharing of best practices across the study team, and regular discussion with the DSMB and study funders regarding operational success and potential thresholds for operational futility.

#### 13.1.1 Recruitment

The sample size of the study is large. The study team will solicit a large number of sites for participation, sufficient to recruit the requisite number of participants and criteria for site selection will include capacity to recruit participants interested in contraceptive services, HIV prevention, and research participation. Each site will develop and revise as needed throughout the enrolment period a plan for recruitment in order to ensure efficient enrolment into the trial. These plans may include a peer to peer recruitment program. Also, on-going community engagement activities will accompany recruitment strategies. The study team recognises that lack of interest in the study question, if present in



CONFIDENTIAL

communities, could result in the potential for slow enrolment; however, recent studies of novel contraceptive methods and HIV prevention technologies have demonstrated high interest.

**13.1.2 Randomisation**

The potential for refusal of women to be randomised among the three trial contraceptive methods is an anticipated risk. The study team will make every effort to counsel women carefully concerning the methods and the process of randomised assignment before randomisation. Considerable evidence exists that many women presenting for contraceptive services do not have a specific method preference and are willing to accept a variety of effective contraceptive methods. Three studies, among women in a variety of settings, have found high willingness to be randomised to a contraceptive method [46, 67]. In addition, a trial in East London, South Africa has successfully recruited and randomised more than 2500 women at risk of HIV infection to either DMPA or copper IUDs[68]. Each of the methods to be evaluated in this study has been found to have high acceptability in a variety of populations, including among African women [35].

**13.1.3 Retention**

High loss to follow-up is an important risk for any clinical trial. Once a participant is enrolled in the trial, study staff will make every effort to retain her in follow-up. Loss to follow-up will be addressed through following ‘best practices’ in retention strategies in prior randomised controlled trials (RCTs) as well as linkages with family planning programs to help women transition after the study. All study sites are responsible for developing and implementing local SOPs to achieve this. Specific components of such procedures are outlined in Section 5.15. The Statistical and Data Management Center will generate monthly reports on the number and percentage of participants completing follow-up visits throughout the course of the study. The study operations team will track retention rates closely and take any required action to address below-target retention rates.

CONFIDENTIAL

## 14. REFERENCES


1. Morrison, C.S., A.N. Turner, and L.B. Jones, *Highly effective contraception and acquisition of HIV and other sexually transmitted infections.* Best Pract Res Clin Obstet Gynaecol, 2009. **23**(2): p. 263-84.
2. Population Reference Bureau. *Family Planning Worldwide 2008 Data Sheet*. 2008; Available from: http://www.prb.org/pdf08/fpds08.pdf.
3. United Nations & Department of Economics and Social Affairs Population Division. *World Contraceptive Use, 2003 Chart*. 2003; Available from: www.un.org/esa/population/publications/contraceptive2003/WCU2003.htm.
4. United Nations, Department of Economics, and S.A.P. Division. *World Contraceptive Use 2011*. 2011; Available from: http://www.un.org/esa/population/publications/contraceptive2011/contraceptive2011.htm.
5. Sullivan, T.M., et al., *Skewed contraceptive method mix: why it happens, why it matters.* J Biosoc Sci, 2006. **38**(4): p. 501-21.
6. Polis, C.B. and K.M. Curtis, *Use of hormonal contraceptives and HIV acquisition in women: a systematic review of the epidemiological evidence.* Lancet Infect Dis, 2013. **13**(9): p. 797-808.
7. Polis, C.B., et al., *An updated systematic review of epidemiological evidence on hormonal contraceptive methods and HIV acquisition in women.* AIDS, 2016. **30**(17): p. 2665-2683.
8. Baeten, J.M., et al., *Hormonal contraceptive use, herpes simplex virus infection, and risk of HIV-1 acquisition among Kenyan women.* AIDS, 2007. **21**(13): p. 1771-7.
9. Heffron, R., et al., *Use of hormonal contraceptives and risk of HIV-1 transmission: a prospective cohort study.* Lancet Infect Dis, 2012. **12**(1): p. 19-26.
10. Morrison, C.S., et al., *Hormonal contraception and HIV acquisition: reanalysis using marginal structural modeling.* AIDS, 2010. **24**(11): p. 1778-81.
11. Wand, H. and G. Ramjee, *The effects of injectable hormonal contraceptives on HIV seroconversion and on sexually transmitted infections.* AIDS, 2012. **26**(3): p. 375-80.
12. Hapgood, J.P., et al., *Not all progestins are the same: implications for usage.* Trends Pharmacol Sci, 2004. **25**(11): p. 554-7.
13. Huijbregts, R.P., et al., *Hormonal contraception and HIV-1 infection: medroxyprogesterone acetate suppresses innate and adaptive immune mechanisms.* Endocrinology, 2013. **154**(3): p. 1282-95.
14. Schindler, A.E., et al., *Classification and pharmacology of progestins.* Maturitas, 2003. **46**: p. 7-16.
15. WHO, *Hormonal contraceptive eligibility for women at high risk of HIV: guidance statement*. 2017, Geneva.
16. Abdool Karim, Q., et al., *Effectiveness and safety of tenofovir gel, an antiretroviral microbicide, for the prevention of HIV infection in women.* science, 2010. **329**(5996): p. 1168-74.
17. Butler, A.R., et al., *Modelling the global competing risks of a potential interaction between injectable hormonal contraception and HIV risk.* AIDS, 2013. **27**(1): p. 105-13.
18. Hatcher, R.A., et al., eds. *Contraceptive Technology*. 20th ed. ed. 2011, Ardent Media, Inc.: Atlanta, GA.
19. Ali, M., J. Cleland, and I. Shah, *Causes and consequences of contraceptive discountinuation: evidence from 60 Demographic and Health Surveys*. 2012, WHO: Geneva.
20. Smit, J.A. and M.E. Beksinska, *Hormonal contraceptive continuation and switching in South Africa: implications for evaluating the association of injectable hormonal contraceptive use and HIV.* J Acquir Immune Defic Syndr, 2013. **62**(3): p. 363-5.
21. Taylor, *Personal communication*.
22. Said, S., et al., *A multicentered phase III comparative clinical trial of depot-medroxyprogesterone acetate given three-monthly at doses of 100 mg or 150 mg: II. The comparison of bleeding patterns. World Health Organization. Task Force on Long-Acting Systemic Agents for Fertility Regulation Special Programme of Research, Development and Research Training in Human Reproduction.* Contraception, 1987. **35**(6): p. 591-610.
23. Kaunitz, A.M., R. Arias, and M. McClung, *Bone density recovery after depot medroxyprogesterone acetate injectable contraception use.* Contraception, 2008. **77**(2): p. 67-76.
24. Power, J., R. French, and F. Cowan, *Subdermal implantable contraceptives versus other forms of reversible contraceptives or other implants as effective methods of preventing pregnancy.* Cochrane Database Syst Rev, 2007(3): p. CD001326.
25. Festin, M., *Personal communication*.


CONFIDENTIAL

26. Meirik, O., et al., *A multicenter randomized clinical trial of one-rod etonogestrel and two-rod levonorgestrel contraceptive implants with nonrandomized copper-IUD controls: methodology and insertion data.* Contraception, 2013. **87**(1): p. 113-20.
27. Kulier, R., et al., *Copper containing, framed intra-uterine devices for contraception.* Cochrane Database Syst Rev, 2007(4): p. CD005347.
28. Stringer, E.M., et al., *A randomized trial of the intrauterine contraceptive device vs hormonal contraception in women who are infected with the human immunodeficiency virus.* Am J Obstet Gynecol, 2007. **197**(2): p. 144-8.
29. Okunlola, M.A., et al., *Discontinuation pattern among IUCD users at the family planning clinic, University College Hospital, Ibadan.* J Obstet Gynaecol, 2006. **26**(2): p. 152-6.
30. Farley, T.M., et al., *Intrauterine devices and pelvic inflammatory disease: an international perspective.* Lancet, 1992. **339**(8796): p. 785-8.
31. Mohllajee, A.P., et al., *Hormonal contraceptive use and risk of sexually transmitted infections: a systematic review.* Contraception, 2006. **73**(2): p. 154-65.
32. Hubacher, D., et al., *Use of copper intrauterine devices and the risk of tubal infertility among nulligravid women.* N Engl J Med, 2001. **345**(8): p. 561-7.
33. Barnhart, K.T., et al., *Risk factors for ectopic pregnancy in women with symptomatic first-trimester pregnancies.* Fertil Steril, 2006. **86**(1): p. 36-43.
34. Morrison, C.S., et al., *Use of sexually transmitted disease risk assessment algorithms for selection of intrauterine device candidates.* Contraception, 1999. **59**(2): p. 97-106.
35. Neukom, J., et al., *Dedicated providers of long-acting reversible contraception: new approach in Zambia.* Contraception, 2011. **83**(5): p. 447-52.
36. Lowe, R.F. and N. Prata, *Hemoglobin and serum ferritin levels in women using copper-releasing or levonorgestrel-releasing intrauterine devices: a systematic review.* Contraception, 2013. **87**(4): p. 486-96.
37. Herkert, O., et al., *Sex steroids used in hormonal treatment increase vascular procoagulant activity by inducing thrombin receptor (PAR-1) expression: role of the glucocorticoid receptor.* Circulation, 2001. **104**(23): p. 2826-31.
38. Hapgood, J.P. and M. Tomasicchio, *Modulation of HIV-1 virulence via the host glucocorticoid receptor: towards further understanding the molecular mechanisms of HIV-1 pathogenesis.* Arch Virol, 2010. **155**(7): p. 1009-19.
39. Koubovec, D., et al., *Synthetic progestins used in HRT have different glucocorticoid agonist properties.* Mol Cell Endocrinol, 2005. **242**(1-2): p. 23-32.
40. Moore, N.L., et al., *An androgen receptor mutation in the MDA-MB-453 cell line model of molecular apocrine breast cancer compromises receptor activity.* Endocr Relat Cancer, 2012. **19**(4): p. 599-613.
41. Kontula, K., et al., *Binding of progestins to the glucocorticoid receptor. Correlation to their glucocorticoid-like effects on in vitro functions of human mononuclear leukocytes.* Biochem Pharmacol, 1983. **32**(9): p. 1511-8.
42. Chandra, N., et al., *Depot Medroxyprogesterone Acetate Increases Immune Cell Numbers and Activation Markers in Human Vaginal Mucosal Tissues.* AIDS Res Hum Retroviruses, 2012.
43. Hel, Z., *Personal communication*, C. Morrison, Editor. 2012.
44. Barousse, M.M., et al., *Susceptibility of middle adolescent females to sexually transmitted infections: impact of hormone contraception and sexual behaviors on vaginal immunity.* Am J Reprod Immunol, 2007. **58**(2): p. 159-68.
45. Morrison, C., Fichrova, R., Mauck, C., Chen, V, Kwok, C., Chipato, T., Salata, R., Doncel, GF. *Biomarkers of cervical inflammation and immunity associated with hormonal contraception, pregnancy and HIV-1 seroconversion*. in *CROI 2012: 19th Conference on Retroviruses and Opportunistic Infections*. 2012. Seattle, WA.
46. Feldblum, P.J., et al., *Randomized assignment to copper IUD or depot-medroxyprogesterone acetate: feasibility of enrollment, continuation and disease ascertainment.* Contraception, 2005. **72**(3): p. 187-91.
47. Gao, J., et al., *Comparison of the clinical performance, contraceptive efficacy and acceptability of levonorgestrel-releasing IUD and Norplant-2 implants in China.* Contraception, 1990. **41**(5): p. 485-94.
48. Haddad, L.B., et al., *Contraceptive adherence among HIV-infected women in Malawi: a randomized controlled trial of the copper intrauterine device and depot medroxyprogesterone acetate.* Contraception, 2013.
49. Alnakash, A.H., *Influence of IUD perceptions on method discontinuation.* Contraception, 2008. **78**(4): p. 290-3.

CONFIDENTIAL

50. Halpern, V., et al., *Strategies to improve adherence and acceptability of hormonal methods of contraception.* Cochrane Database Syst Rev, 2011(4): p. CD004317.
51. Canto De Cetina, T.E., P. Canto, and M. Ordoñez Luna, *Effect of counseling to improve compliance in Mexican women receiving depot-medroxyprogesterone acetate.* Contraception, 2001. **63**(3): p. 143-146.
52. Nanda, K., et al., *Discontinuation of oral contraceptives and depot medroxyprogesterone acetate among women with and without HIV in Uganda, Zimbabwe and Thailand.* Contraception, 2011. **83**(6): p. 542-8.
53. Winner, B., et al., *Effectiveness of long-acting reversible contraception.* N Engl J Med, 2012. **366**(21): p. 1998-2007.
54. Peipert, J.F., et al., *Continuation and satisfaction of reversible contraception.* Obstet Gynecol, 2011. **117**(5): p. 1105-13.
55. Nanda, K., *Personal communication.* 2013.
56. UNAIDS, *Global Report Fact Sheet: Sub-Saharan Africa*. 2010: Geneva.
57. UNAIDS, *Global Report: UNAIDS Report on the Global AIDS Epidemic 2012*. 2012, UNAIDS: Geneva.
58. UNAIDS, *World AIDS Day Report 2012*. 2012, UNAIDS: Geneva.
59. Van Damme, L., et al., *Preexposure prophylaxis for HIV infection among African women.* N Engl J Med, 2012. **367**(5): p. 411-22.
60. Johnson, L.F., et al., *The effect of changes in condom usage and antiretroviral treatment coverage on human immunodeficiency virus incidence in South Africa: a model-based analysis.* J R Soc Interface, 2012. **9**(72): p. 1544-54.
61. WHO, *Selected practice recommendations for contraceptive use*. 2nd ed. 2004, Geneva: World Health Organization.
62. Tepper, N.K., et al., *Retention of intrauterine devices in women who acquire pelvic inflammatory disease: a systematic review.* Contraception, 2013. **87**(5): p. 655-60.
63. Sufrin, C.B., et al., *Neisseria gonorrhea and Chlamydia trachomatis screening at intrauterine device insertion and pelvic inflammatory disease.* Obstet Gynecol, 2012. **120**(6): p. 1314-21.
64. WHO, *Update to selected practice recommendations for contraceptive use*. 2008, Geneva: World Health Organization.
65. World Health Organization (WHO), *Medical eligibility criteria for contraceptive use*. 4th ed. 2010, Geneva: WHO.
66. AVAC, U., *Good participatory practice guidelines for biomedical HIV prevention trials*. 2011.
67. Hubacher, D., et al., *Hormonal contraception and the risks of STI acquisition: results of a feasibility study to plan a future randomized trial.* Contraception, 2008. **77**(5): p. 366-70.
68. Hofmyer, J., *Personal communication*. 2012.


CONFIDENTIAL

## 15. APPENDICES

**Appendix 1: Membership and roles for the organizations comprising the ECHO Consortium**

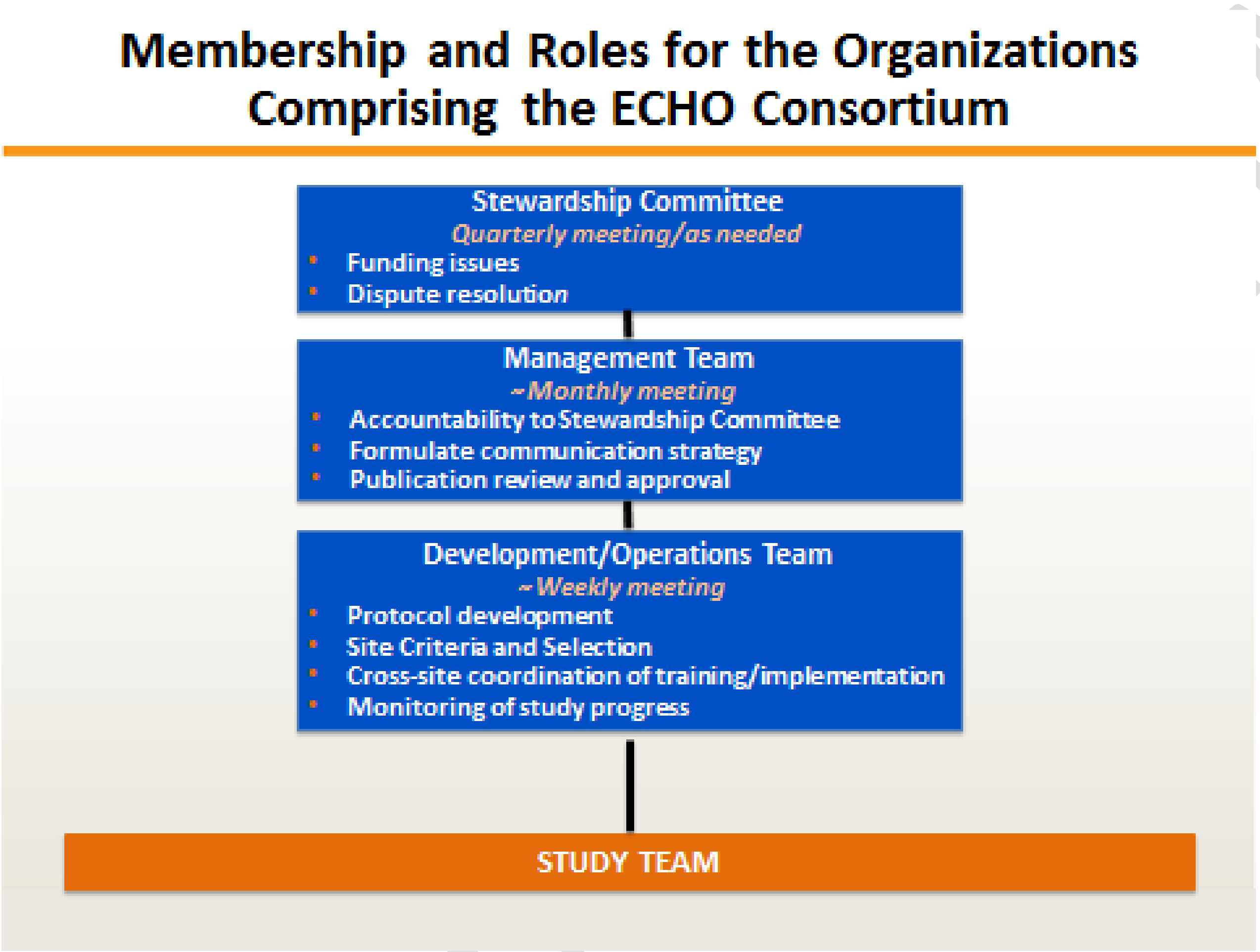

## Appendix 2: ECHO Study Sites

KEMRI-RCTP Study Centre, Lumumba Health Centre
*Kisumu, Kenya*

Effective Care Research Unit at Cecilia Makiwane Hospital
*East London, South Africa*

MatCH Research Unit Commercial City Clinic
*Durban, South Africa*

MatCH Research Unit Edendale Clinical Research Site
*Pietermaritzburg, South Africa*

Setshaba Research Centre
*Soshanguve, South Africa*

The Aurum Institute
*Klerksdorp, South Africa*

Madibeng Centre for Research
*Brits, South Africa*

Emavundleni Research Centre
*Cape Town, South Africa*

Wits Reproductive Health and HIV Institute (WRHI)
*Johannesburg, South Africa*

Family Life Association of Swaziland (FLAS)
*Mbabane, Swaziland*
*Manzini, Swaziland*

UNC Global Projects Zambia/Kamwala Clinic
*Lusaka, Zambia*

Qhakaza Mbodoko Research Clinic
*Ladysmith, South Africa*

CONFIDENTIAL

## Appendix 3: Three-arm study design: What will it answer?

| DMPA-Implant-IUD | DMPA-NET-En-Implant | DMPA-NET-En-IUD |
|---|---|---|
| ▪ Provides evidence of the effect of DMPA vs. two comparators (potentially providing some sense of whether, in the case of differences in HIV, comparators decrease risk or DMPA increases risk).<br>▪ Differences in discontinuation rates among the three methods (including among young women).<br>▪ Whether DMPA increases HIV risk above a specified level of effect compared to a low-dose highly effective HC.<br>▪ Whether DMPA increases HIV risk compared to a non-hormonal, highly-effective method.<br>▪ Whether there is any difference in HIV risk between the IUD and a low-dose implant.<br>▪ Differences in pregnancy rates between a short-acting progestin-only contraceptive and two long-acting methods. | ▪ Provides evidence of the effect of DMPA vs. two comparators (potentially providing some sense of whether, in the case of differences in HIV, comparators decrease risk or DMPA increases risk).<br>▪ Differences in discontinuation rates among the three methods (including among young women).<br>▪ Whether DMPA increases HIV risk above a specified level of effect compared to a low-dose highly effective HC (i.e., implant).<br>▪ Whether DMPA increases HIV risk compared to a different progestin-only injectable contraceptive.<br>▪ Whether there is any difference in HIV risk between NET-En and a low-dose implant.<br>▪ Differences in pregnancy rates between two short-acting progestin-only contraceptives and an implant.<br>▪ Whether women discontinuing DMPA are more likely to switch to another injectable or another type of method. | ▪ Provides evidence of the effect of DMPA vs. two comparators on HIV risk (providing some sense of whether, in the case of differences in HIV, NET-En or IUD decrease risk or DMPA increases risk).<br>▪ Differences in discontinuation rates among the three methods (including among young women).<br>▪ Whether DMPA increases HIV risk above a specified level of effect compared to a non-hormonal highly effective method (i.e., IUD).<br>▪ Whether DMPA increases HIV risk compared to a different progestin-only injectable contraceptive.<br>▪ Whether there is any difference in HIV risk between NET-En and IUD.<br>▪ Differences in pregnancy rates between two short-acting progestin-only contraceptives and the IUD.<br>Whether women discontinuing DMPA are more likely to switch to another injectable as opposed to another type of method. |
| ▪ Difference in HIV risk between DMPA and another progestin-only injectable method.<br>▪ Not definitively answer whether any DMPA effect is limited to the 'true biological effect'.<br>▪ If a difference in HIV incidence is seen between the other arms and the IUD arm, interpretation could be that the IUD decreases HIV risk (compared to a null of no contraception), rather than the others increasing risk. Nonetheless, the study would still answer the question about the *relative* HIV risk across the three | ▪ Cannot answer question about difference in HIV risk between DMPA and a non-hormonal method (e.g., copper IUD).<br>▪ Not definitively answer whether any DMPA effect is limited to the 'true biological effect'– a limitation of all trial designs.<br>▪ Whether estrogenic containing methods have a protective effect. | ▪ Difference in HIV risk between DMPA and low-dose, long-acting hormonal methods (e.g., implants).<br>▪ If a difference in HIV incidence is seen between the injectable arms and the IUD arm, one interpretation could be that the IUD decreases HIV risk (compared to a null of no contraception), rather than the injectables increasing risk. Nonetheless, the study would still answer the question about the *relative* HIV risk across the three contraceptive methods (i.e., the public health question – if contraception is desired, what is the HIV risk for one method vs. another). |

CONFIDENTIAL

| | | |
|---|---|---|
| contraceptive methods (i.e., the public health question – assuming contraception is desired, what is the HIV risk for one type vs. another).<br>▪ Whether estrogen containing methods are protective.<br>▪ Whether women discontinuing DMPA are more likely to switch to another injectable or to another type of method. | | ▪ Not definitively answer whether a DMPA effect is limited to a 'true biological effect'.<br>▪ Whether estrogen-containing methods have a protective effect. |

CONFIDENTIAL

## Appendix 4: Advantages and Disadvantages of Specific Contraceptive Methods as a Study Arm

| ARM | DMPA | NET-En | IMPLANT | IUD (copper) | COCs | Combined Injectable |
|---|---|---|---|---|---|---|
| Reasons to include in a multi-arm RCT | ▪ Method most strongly implicated with HIV risk<br>▪ Broad consensus that additional data are necessary to support or refute a DMPA-HIV association<br>▪ Discrete (hidden)<br>▪ Number of MPTs are considering using MPA – must understand risk.<br>▪ Method most widely used in SSA, HIV prevention trials<br>▪ Method pushed for community-based distribution in many countries<br>Method used in Uniject technology | ▪ Injectable method; potentially easier transition should DMPA be associated with elevated HIV risk.<br>▪ Widely used in South Africa – esp. in young women<br>▪ Different progestin than DMPA<br>▪ Discrete (hidden)<br>▪ Little data available | ▪ Lower-dose hormonal method<br>▪ Different progestin than DMPA<br>▪ Long-acting method<br>▪ Low discontinuation and pregnancy rates; highly effective in typical use<br>▪ Use increasing rapidly in some SSA countries<br>▪ Discrete, although not necessarily fully hidden | ▪ Non-hormonal method<br>▪ Long-acting method<br>▪ Low pregnancy rates; highly effective in typical use<br>▪ Discrete<br>▪ Low-cost over 10 years<br>▪ Possible protective effect against HIV (associated with copper) | ▪ Easy to dispense<br>▪ Mostly widely used hormonal method worldwide<br>▪ Widely used in some SSA countries (e.g. Zimbabwe)<br>▪ No upfront costs for equipment or personnel<br>▪ More predictable bleeding patterns | ▪ Combined (E&P) method<br>▪ Injectable method; thus potentially an easier transition should DMPA be associated with elevated HIV risk<br>▪ More predictable bleeding patterns<br>▪ Discrete/hidden |

CONFIDENTIAL

| ARM | DMPA | NET-En | IMPLANT | IUD (copper) | COCs | Combined Injectable |
|---|---|---|---|---|---|---|
| Reasons not to include in a multi-arm RCT | ▪ Can be high discontinuation – a priority must be to minimise discontinuation | ▪ Not a low-dose method<br>▪ Not long-acting<br>▪ Higher discontinuation/pregnancy rates in typical use than IUD/implants<br>▪ Not widely used in SSA or worldwide<br>▪ Not widely registered/available | ▪ Requires equipment and training, but can be inserted by low level health care worker<br>▪ Not widely known/accepted by women in some settings<br>▪ Not registered or available in some countries<br>▪ Up-front costs for system/client | ▪ Requires equipment and training<br>▪ Not used widely in SSA<br>▪ Up-front costs for system/client<br>▪ Barriers to insertion/use in nulliparous women especially in younger teenagers | ▪ Not a low-dose method<br>▪ Not a long-acting method<br>▪ Higher discontinuation/pregnancy rates in typical use (and in HIV prevention trials)<br>▪ Often poor adherence<br>▪ Difficult to measure adherence<br>▪ Many more contraindications than progestin-only contraceptives (POCs) | ▪ Not a low-dose method<br>▪ Some contain same progestin as DMPA<br>▪ Requires monthly visits<br>▪ Not a long-acting method<br>▪ Higher discontinuation-pregnancy rates in typical use<br>▪ Not registered or used in SSA<br>▪ Many more contraindications than POCs |

CONFIDENTIAL

**Appendix 5: Site Evaluation Metrics**

| | |
|---|---|
| **Population** | |
| HIV incidence | • Cross-site incidence ~3.5% annually<br>    o individual sites incidence at least 2.0-2.5% from recent incidence estimates (ideally from last 3 years)<br>    o direct measures of incidence (if direct measures not available, reliable prevalence measures considered) |
| Clinic location and target population | • Proposed location of the study clinic, including use of clinic vs. new location<br>• Demographics of population attending the clinic (age, seeking family planning services, etc.)<br>• Experience recruiting and access to specific subpopulations (post-partum, post-abortion, young women [under 25 years]) |
| **Infrastructure** | |
| Clinic | • Status of current space and staffing<br>• Ability to accommodate research study of this size<br>• Need for renovations or equipment prior to study initiation<br>• Experience and QA for rapid HIV testing. |
| Laboratory | • Proposed laboratory<br>• Ability to conduct study assays (HIV serologic confirmatory testing, CD4 counts, plasma HIV RNA, STI NAAT) and current QA procedures |
| **Contraception** | |
| Contraceptive experience | • Expertise of the research staff in delivering the contraceptive methods in the protocol (injections, implants, IUDs) and treating, or referring for, complications |
| Method use | • Current distribution of family planning methods among clients<br>• Numbers of women initiating study methods per month |
| **HIV** | |
| HIV experience | • Investigator and research team experience with HIV service delivery |
| Referral options | • Local availability of referral (within or outside organization) for HIV care for women diagnosed with HIV (required) |
| **Research** | |
| Research experience | • Contraceptive research experience<br>• HIV research experience<br>• Ability to conduct randomised trials and retain subjects in prospective studies lasting 18 months<br>• Other clinical trial experience (details of study reports and summaries of GCP audits or monitoring reports, if available) |
| PI/I of Record | • Experience, availability, and motivation of the proposed site PI/investigator |
| **Regulatory** | |
| IRB | • Availability of site IRB<br>• Site IRB meeting schedule<br>• IRB membership and procedures<br>• Other regulatory requirements (e.g. national science council, national drug regulatory authority) |
| Specimens | • Requirements for international shipment (within region and/or to the US) of specimens |
| Products | • Licensure status of the relevant study products |

CONFIDENTIAL

## Appendix 6: Schedule of study procedures

| | SCR | ENR | MTH 1 | QTRYLY | FINAL | POSSIBLE SEROCONVERSION |
|---|---|---|---|---|---|---|
| **ADMINISTRATIVE AND REGULATORY** | | | | | | |
| Written informed consent (include future/addt'l testing) | X | X[1] | | | | |
| Assignment of participant identification (PTID) numbers | X | | | | | |
| Assessment/ confirmation of eligibility | X | X | | | | |
| Collection/review/update of locator information | X | X | X | X | X | X |
| Participant reimbursement | X | X | X | X | X | X |
| Visit scheduling | X | X | X | X | | X |
| **CONTRACEPTIVE** | | | | | | |
| Contraceptive counselling, including continuation counselling | X | X | X | X | X | X |
| Randomisation and first injection, implant/IUD insertion | | X | | | | |
| Contraceptive side effects assessment | | | X | X | X | X |
| Injectable contraceptive provision | | X | | X | X | X |
| **BEHAVIOURAL** | | | | | | |
| Risk reduction counselling | X | X | | X | X | X |
| HIV pre- and post-test counselling | X | | | X | X | X |
| Behavioural data collection | | X | | X | X | X |
| **CLINICAL** | | | | | | |
| Demographic history | X | | | | | |
| Contraceptive, reproductive history/update | X | X | | X | X | X |
| Physical examination (height, weight, BP) | X | | | * | * | X |
| Pregnancy assessment | X | | | X | | |
| Pelvic examination (including bimanual and/or speculum as indicated and defined in the MOP) | X | IUD only | | * | X | X |
| IUD string check (confirmation by ultrasound if strings not found) | | | X | | X | X |

CONFIDENTIAL

| | SCR | ENR | MTH 1 | QTRYLY | FINAL | POSSIBLE SEROCONVERSION |
|---|---|---|---|---|---|---|
| Screen for cervical cancer (optional –based on local standards of care and availability of treatment) | X | | | | | |
| Syndromic assessment for STIs | X | * | | * | X | X |
| AE assessment | | | X | X | X | X |
| **LABORATORY** | | | | | | |
| Urine pregnancy test | * | X^ | | * | X^ | X^ |
| HIV rapid test | X | | | X | X | X |
| HIV confirmatory testing (HIV Western blot and/or HIV EIA, with HIV RNA PCR) | | | | | | X |
| HIV RNA and CD4 count+ | | | | X | X | X |
| Endocervical swab for STI testing (NAAT for GC, CT) | X | | | | X | X |
| Endocervical swab for archiving | X | | | | X | X |
| Cervicovaginal specimen for semen exposure testing | | | | | X | X |
| Plasma and Serum archive (for HIV and HSV-2 testing) # | | X | | X (6M only) | X | X |

[1] Only for sites which opt to use separate screening and enrolment consent forms
*as clinically indicated;
^if a woman has had a clinician verified miscarriage or pregnancy termination within 12 days, urine pregnancy test is not needed
# HSV-2 testing will be done on enrolment specimens; testing at other visits dependent on additional funding
+ HIV RNA and CD4 count will be done for confirmed seroconverters

Notes:

- Any visit (screening, enrolment, follow-up, final, seroconversion) can be followed by an as-needed visit to provide laboratory test results (e.g., STI, confirmatory HIV, etc.) that return after that visit and any related counselling and treatment.
- Confirmed HIV-positive women will continue to be followed on the study schedule, with all procedures except on-going HIV serologic testing. HIV RNA and CD4 counts will be done for seroconverters at the time of seroconversion, at subsequent quarterly visits, and at the final study visit. In addition, the enrolment plasma archive sample will be tested by HIV RNA PCR for all HIV seroconverters to define whether seroconverters were infected at the time of enrolment, as described in Section 8.2.1
- SCR = screening; ENR = enrolment; FINAL VISIT = last on-study visit, to permit completion of procedures in the event of early participant or study termination; POSSIBLE SEROCONVERSION = potential HIV seroconversion (i.e., visit at which rapid testing is positive, while confirmatory testing pending)

CONFIDENTIAL

## Appendix 7: HIV Testing Algorithm

**Proposed algorithm** **Parallel rapid tests**

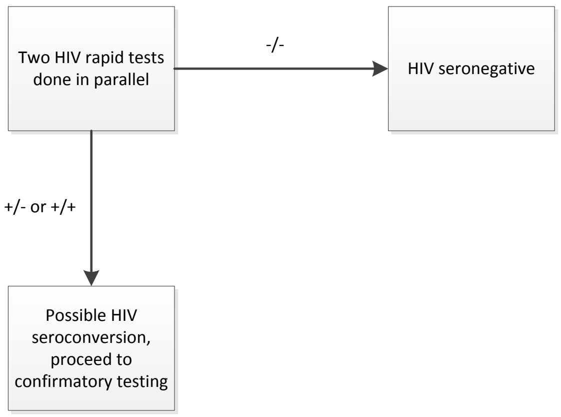


**Confirmed HIV seroconversion** = positive HIV results as determined by the Endpoints Committee. In the event of possible or confirmed HIV seroconversion, follow-up will continue and randomised contraceptive method need not be stopped/discontinued.

CONFIDENTIAL

## Appendix 8: Additional sample collection for ancillary studies of biologic markers of early HIV disease

| | ENR | MONTH 6 | FINAL VISIT | POSSIBLE SEROCONVERSION | QUARTERLY AFTER SEROCONVERSION |
|---|---|---|---|---|---|
| **LABORATORY** | | | | | |
| Genital swab archive (for studies of mucosal immunology and microbiology) | | X | X | X | |
| Plasma archive (for studies of contraceptive pharmacokinetics, immunologic factors related to HIV risk and virologic studies in HIV seroconverters) | X | X | X | X | X |
| Whole blood archive (for genomic factors related to HIV risk) | X | | | | |
| Peripheral blood mononuclear cells (for functional immunologic factors related to HIV risk) | X | X | | X | |
| HIV RNA and CD4 (for more detailed studies of early HIV disease) | | | | | X |

CONFIDENTIAL

ENR = enrolment; FINAL VISIT = last on-study visit, to permit completion of procedures in the event of early participant or study termination; POSSIBLE SEROCONVERSION = potential HIV seroconversion (i.e., visit at which rapid testing is positive, while confirmatory testing pending)

If not obtained at screening/enrolment, consent for additional sample collection can be obtained at any subsequent visit.

**Appendix 9: Additional sample collection for ancillary studies of biologic correlates of HIV acquisition***

| | SCR | ENR | MONTHS 1, 3, 6, 9, 12, 15, 18 | FINAL VISIT | POSSIBLE SEROCONVERSION |
|---|---|---|---|---|---|
| Vaginal swab archive (for studies of mucosal immunology, microbiology and hormone concentrations) | X | X | X | X | X |
| Endocervical swab archive (for studies of mucosal immunology, microbiology and hormone concentrations) | X | X | X | X | X |
| Cytobrush archive (for studies of mucosal immunology, microbiology and hormone concentrations) | X | X | X | X | X |
| Softcup (to collect cervical exudate for studies of mucosal immunology, microbiology and hormone concentrations) | X | X | X | X | X |
| Whole blood archive (for studies of immunology and hormone concentrations) | X | X | X | X | X |

* Specimens outlined in this table are for ancillary studies and are not part of the specimen collection

CONFIDENTIAL

for the main ECHO Trial.  Participants may agree to have their regular ECHO Study specimens stored for further testing but decline to have specimens taken and stored for ancillary studies.

ENR = enrolment; FINAL VISIT = last on-study visit, to permit completion of procedures in the event of early participant or study termination; POSSIBLE SEROCONVERSION = potential HIV seroconversion (i.e., visit at which rapid testing is positive, while confirmatory testing pending)

If not obtained at screening/enrolment, consent for additional sample collection can be obtained at any subsequent visit.

CONFIDENTIAL

**Appendix 10: Medications that may cause drug interactions with some study methods**

**Antiretrovirals**

- Protease inhibitors
- NNRTIs

**Antimycobacterials**

- rifampicin
- rifabutin
- rifapentine

**Anticonvulsants**

- carbamazepine
- oxcarbazepine
- phenobarbital (phenobarbitone)
- phenytoin
- primidone
- topiramate

**Anxiolytics/Hypnotics/ Sedatives**

- midazolam
- propofol
- triazolam
- alprazolam

**Immunosuppressants**

- cyclosporin
- sirolimus
- tacrolimus

**Oral hypoglycemics**

- pioglitazone
- rosiglitazone

**Others:**

- St. John's Wort (herbal)
- modafinil (Provigil) (stimulant)
- lansoprazole (proton pump inhibitor)

CONFIDENTIAL

## Appendix 11: ECHO Governance

**Stewardship Committee**

- Assure financial resources
- Assure operational milestones of trial are met

**Management Committee**

- Provide accountability to Stewardship Committee
- Coordinate funding among different organizations and sites
- Oversee timelines for major trial milestones
- Coordinate ECHO team meetings
- Interface with the DSMB
- Set priorities for trial publications Approve final site selection and oversee decisions relating to site continuation
- Act as a moderator in resolving disputes between key stakeholders in the committee structures
- Act as the 'face' of the study and undertake relevant communications activities
- Direct reports include: Protocol Design Committee, Protocol Implementation Committee, Site Selection Committee, and Communications Committee

**Protocol Design Committee**

- Considers all design issues in preparing the ECHO protocol
- Responds to evaluations by external review panels
- Provides design responses to reactions from IRBs
- Reports to Management Committee

**Protocol Implementation Committee**

- Responsible for monitoring progress of the trial and producing and reviewing monthly reports
- Responsible for giving monthly trial feedback, to each of the sites and offering them suggestions for corrective action
- Oversee Subcommittees
- Coordinate and track IRB submissions
- Oversee and triage site communications
- GPP Plan
- Write SOPs
- Manage trial data
- Coordinate site training
- Oversee site performance
- Ensure adherence to GCP



CONFIDENTIAL

- Oversee clinical monitoring contractors
- Oversee development of appropriate repository and sample collection systems
- Coordinate external laboratory testing
- Appoint and oversee appropriate international laboratory oversight group
- Develop and review SOPs for laboratory procedures
- Develop laboratory plan for trial
- Reports to Management Committee

**Protocol Team Call**

- Monthly call for sharing of best practices
- Site-to-site and site-to-core sharing of progress, issues and ideas
- All team review of progress and potential solutions
- Opportunity for sites to present and discuss broad trial issues/ideas
- Sharing of operational guidance

**CRF/Data Management Subcommittee**

- Develop CRFs
- Develop trial database
- Develop DM related SOPs
- Train on DM
- Reports to Protocol Implementation Committee

**Communication Committee**

- Develop and implement trial communications plan
- Develop appropriate tools for dispersing trial information for internal and external stakeholders
- Develop appropriate tools for communication about the trial to participants, and development of motivational materials for the participants
- Fulfill all communications requirements as outlined in GPP / GPP communications guidelines
- Convene and oversee appropriate local and international stakeholder meetings
- Reports to Management Committee

**Site Selection Committee**

- Develop site selection criteria
- Conduct site assessment visits
- Select sites to participate in trial
- Will hibernate after initial site selection & reconvened, if required, if/when additional sites required
- Reports to Management Committee

**Contraceptive Training, Management and Safety Subcommittee**



CONFIDENTIAL

- Draft training SOPs, manuals for insertion, monitoring and/or removal of contraceptives to be used in trial
- Provide contraceptive training as needed for study clinicians/nurses
- Provide oversight and guidance for contraceptive use during trial
- Contraceptive continuation materials development
- Coordinate a safety review team to review and report on SAEs as well as review method related adverse events
- Develop safety monitoring plan
- Reports to Protocol Implementation Committee

**Contraceptive Procurement & Distribution Subcommittee**

- Assess ability to procure trial contraceptives from different sources
- Determine which brand (if multiple options available) of contraceptive to procure for trial
- Coordinate any contraceptive product testing necessary for the trial
- Determine ability to receive donations versus purchase contraceptives
- Coordinate shipments and oversee quantities of contraceptives
- Ensure sufficient quantities of contraceptives available for sites
- Appoint clinical supplies manager
- Provide centralized location for storage and shipment of contraceptives to trial sites
- Oversee quantities shipped to sites and requests for additional supplies
- Ensure appropriate storage conditions for products
- Reports to Protocol Implementation Committee

**Endpoints Subcommittee**

- Establish Endpoints Committee and processes related to endpoints review
- Reports to Protocol Implementation Committee

CONFIDENTIAL
